# Supplementary material for: The rise of grasslands is linked to atmospheric CO2 decline in the late Palaeogene
Source: Nat Commun. 2022 Jan 12;13:293. doi: 10.1038/s41467-021-27897-y (PMC8755714; doi:10.1038/s41467-021-27897-y)
Supplement: Supplementary file 1 — Supplementary Information [file 41467_2021_27897_MOESM1_ESM.pdf]

The rise of grasslands is linked to atmospheric CO_2 decline in the late Palaeogene

Supporting Information

Luis Palazzesi[1,2,*], Oriane Hidalgo[2,3], Viviana D. Barreda[1], Félix Forest[2] and Sebastian Höhna[4,5,**]

[1] *Museo Argentino de Ciencias Naturales & Consejo Nacional de Investigaciones Científicas y Técnicas (CONICET), Buenos Aires C1405DJR, Argentina*
[2] *Jodrell Laboratory, Royal Botanic Gardens, Kew, Richmond, Surrey TW9 3DS, United Kingdom*
[3] *Institut Botànic de Barcelona (IBB, CSIC-Ajuntament de Barcelona), Catalonia, Spain*
[4] *GeoBio-Center, Ludwig-Maximilian-Universität München, 80333 Munich, Germany*
[5] *Department of Earth and Environmental Sciences, Paleontology & Geobiology, Ludwig-Maximilian-Universität München, 80333 Munich, Germany*

Correspondence should be sent to:
∗ lpalazzesi@macn.gov.ar
∗∗ hoehna@lmu.de

Contents

S1 Taxonomic representativeness of the most important vascular plant families in grasslands . . 3
S2 Extent of major biomes (107 km^2) using an equilibrium vegetation model (BIOME4) 4
S3 Asteraceae fossil species 5
S4 Asteraceae genes 6
S5 Poaceae genes 7
S6 Environmental CO_2 curve 8
S7 Models and Priors for the environmental correlation analyses 9
S7.1 Fixed 9
S7.2 UC 9
S7.3 Gaussian Markov Random Field (GMRF) 10
S7.4 HSMRF 10
S8 Diversification rates with phylogenies 12
S8.1 Diversification shifts against simplified phylogenies 12
S8.2 Diversification shifts among lineages 13
S9 The effect of different prior models on diversification rates 14
S10 The effect of number of epochs on diversification rates 15
S11 Model selection of diversification models 16
S12 The effect of different taxon sampling methods 17
S13 The effect of different models on correlations to the environmental variable 18
S14 Model selection of environmental variable correlated models 19
S15 The effect of binning on environmental variable correlated models 20
S16 The effect of node calibration on environmental variable correlated models 21

S17 The effect of environmentally-dependent diversification models on diversification rates 22
S18 Simulation study of environmentally-dependent diversification rates 24
S18.1 Simulated diversification rates under environmentally-dependent diversification rates models 24
S18.2 Simulated phylogenies under environmentally-dependent diversification rates 26
S18.3 Estimated correlation factors from the phylogenies simulated under environmentally-dependent diversification rates 28
S19 Simulation study using empirical taxon sampling 30

S1 Taxonomic representativeness of the most important vascular plant families in grasslands

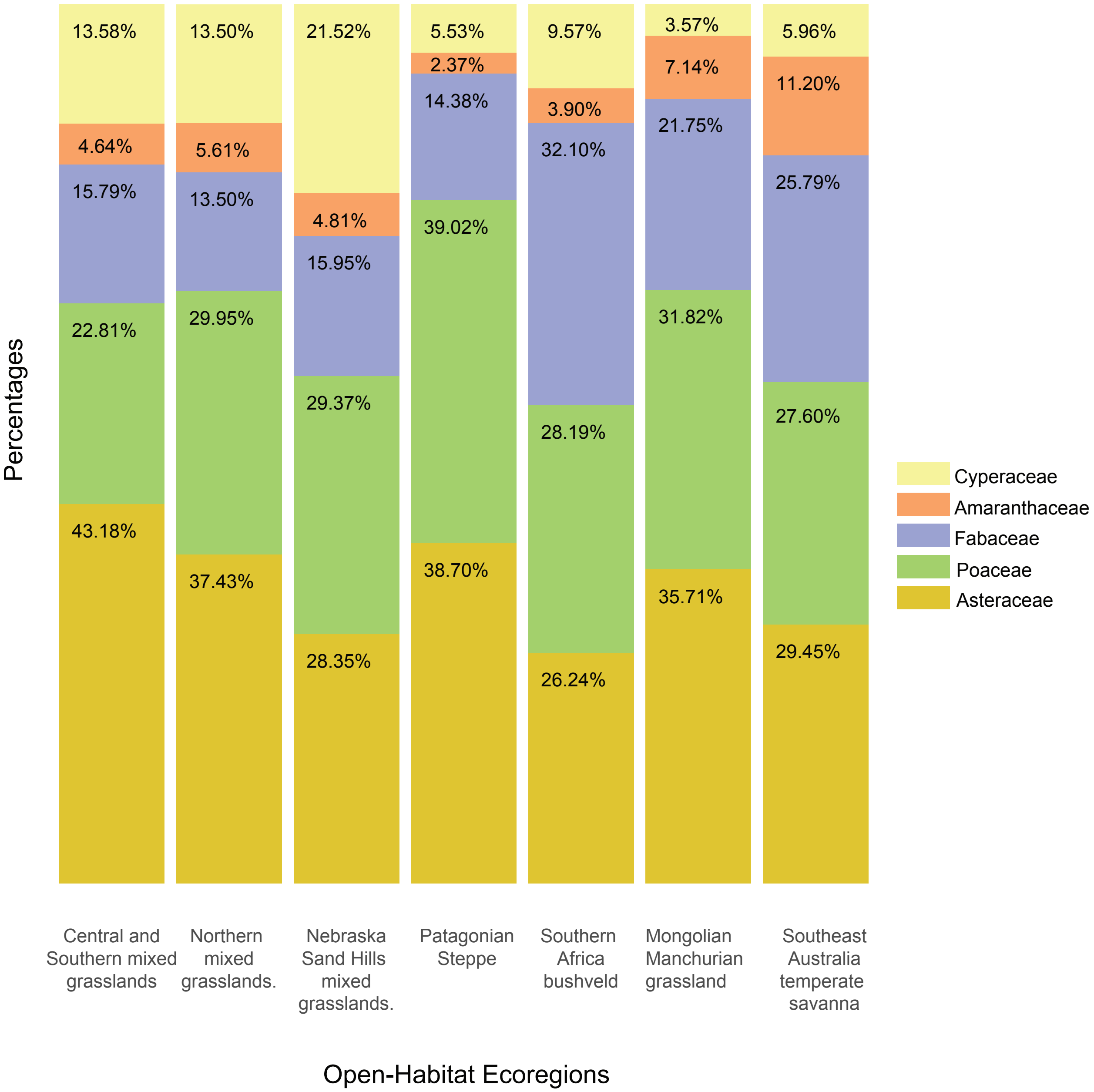

Figure S1: Taxonomic representativeness of the most important vascular plant families in grasslands. We selected seven WWF open-habitat eco-regions and quantified the number of species of the most abundant families using the R package ‘rgbif’ [5].

S2 Extent of major biomes (107 km^2) using an equilibrium vegetation model (BIOME4)

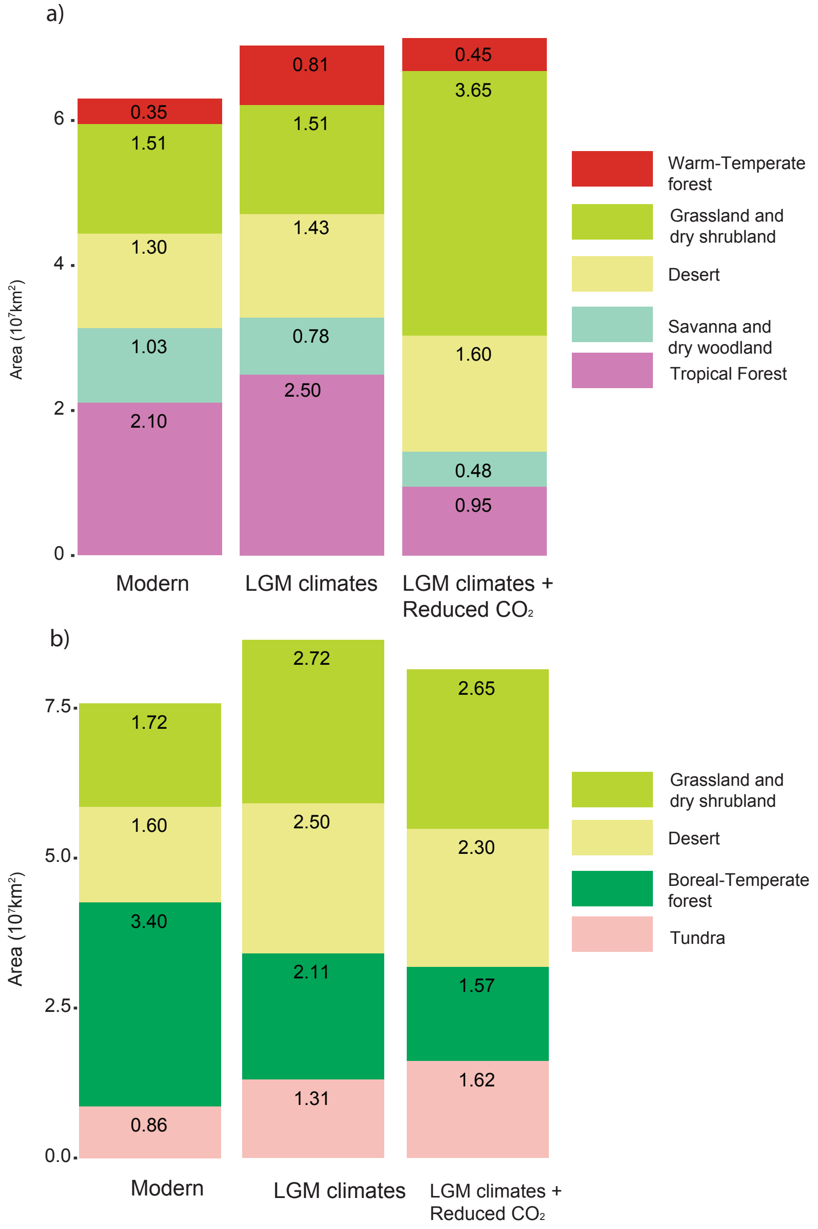

Figure S2: Extent of major biomes (107 km^2) using an equilibrium vegetation model (BIOME4) [9]. a) Intertropical (30°N-30°S) extent of tropical forest, savanna and dry woodland, desert, grassland and dry shrubland and warm-temperate forest as a result of simulated LGM climate changes alone, LGM climate and reduced CO_2 concentrations compared to modern extent of the same biomes. b) Northern hemisphere extent of tundra, boreal-temperate forest, desert and grassland and dry shrubland as a result of simulated LGM climate changes alone, LGM climate plus reduced CO_2 conditions. The output of each of the seventeen climate simulations was averaged for each major biome in order to obtain a more compelling story about the effects of climate and CO_2 concentrations on the vegetation. Overall, the modeled physiological impact of low CO_2 produces i) a pronounced shift towards open-habitat vegetation, and ii) a reduction of ~50% of reduction in the area of tropical forests compared to the area simulated under modern climate and CO_2 conditions [9].

S3 Asteraceae fossil species

Table S1: Asteraceae fossil species. Fossil data from Asteraceae come almost exclusively from pollen grains. The oldest Asteracean-like pollen comes from the latest Cretaceous of Antarctica and New Zealand [3], assigned to the Barnadesioideae, the sister subfamily to the core-Asteraceae. The unique macrofossil (inflorescence and associated pollen grains) confidently assigned to Asteraceae comes from the Middle Eocene of Patagonia showing similarities with the Mutisioideae and Carduoideae [2]. The earliest record of the Asteroideae (the clade that includes the most common open-habitat daisy tribes) occurs since the Late Oligocene of New Zealand but in very low frequencies. Fossils refer to this subfamily increased in abundance and diversity during the Miocene and Pliocene. Pollen referred to *Artemisia*, in particular, did not become abundant until the Middle-Late Miocene with several reports from central Europe, Asia and North America. Pre-Miocene findings need further verification. Overall, the Late Oligocene and in particular the Miocene witnessed the major step on the diversification of Asteraceae; ca. 80% of the fossil species recorded have been defined for this time interval.

Species	Botanical Affinity	Epoch
Tubulifloridites lilliei type A *sensu* Barreda et al [3]	Barnadesioideae	Maastrichtian
Tubulifloridites sp. *sensu* Raine 2008 [16]	Mutisioideae/Carduoideae?	Early Eocene
Mutisiapollis tellerieae Barreda and Palazzesi	Mutisioideae/Carduoideae	Middle Eocene
Raiguenrayun cura Barreda et al	Mutisioideaea/Carduoideae	Middle Eocene
Mutisiapollis patersonii Macphail and Hill	Mutisioideae (Mutisieae, *Mutisia* type)	Early Oligocene
Tubulifloridites antipodica Cookson	Asteroideae	Oligocene/Miocene
Tricolporopollenites microechinatus Trevisan	Asteroideae	Oligocene/Miocene
Mutisiapollis viteauensis Barreda	Gochnatioideae (*Cnicothamnus* type)	Early Miocene
Huanilipollis cabrerii Barreda and Palazzesi	Mutisioideae (Nassauvieae)	Early Miocene
Huanilipollis criscii Barreda and Palazzesi	Mutisioideae (Nassauvieae)	Early Miocene
Cichorieacidites ixeriformis Zheng	Cichorioideae	Middle Miocene
Lapsana type Lancucka-Srodoniowa	Cichorioideae (Cichorieae)	Middle Miocene
Tubulifloridites granulosus Nagy	Asteroideae	Miocene
Tubulifloridites simplis Martin	Asteroideae	Miocene
Echitricolporites spinosus Germeraad et al	Asteroideae	Miocene
Tubulifloridites anthemidearum Nagy	Asteroideae (Anthemideae)	Miocene
Artemisia type *sensu* Leopold [14]	Asteroideae (Anthemideae)	Miocene
Tubulifloridites ambrosinae Nagy	Asteroideae (Ambrosiinae)	Miocene
Tubulifloridites sp. (*Ambrosia* type) Guler et al	Asteroideae (Heliantheae)	Miocene
Xanthium type Cavallo and Martinetto	Asteroideae (Heliantheae)	Miocene
Tubulifloridites grandis Nagy	Asteroideae?	Miocene
Compositae indet *sensu* Graham [8]	Asteroideae?	Miocene
Quilembaypollis gamerroi Palazzesi and Barreda	Barnadesioideae (*Chuquiraga type*)	Miocene
Quilembaypollis tayuoides Barreda and Palazzesi	Barnadesioideae (*Dasyphyllum* type)	Miocene
Quilembaypollis stuessyi Palazzesi and Barreda	Barnadesioideae (*Schlechtendalia* type)	Miocene
Cirsium type Lancucka-Srodoniowa	Carduoideae	Miocene
Cichoriaearumpollenites gracilis Nagy	Cichorioideae	Miocene
Cichorium intybus type Hochuli	Cichorioideae (Cichorieae)	Miocene
Mutisiapollis sp. *sensu* Barreda et al 2006 [1]	Mutisioideae (Mutisieae)	Miocene
Tricolporopollenites microspinulitegillatus Trevisan	Asteroideae (Anthemideae, *Artemisia* type)	Miocene/Pliocene
Tricolporopollenites rarispinulitegillatus Trevisan	Asteroideae (Anthemideae, *Artemisia* type)	Miocene/Pliocene
Artemisiaepollenites minor Zhu	Asteroideae (Anthemideae)	Miocene/Pliocene
Tubulifloridites minutus Regali	Asteroideae (Astereae, *Solidago* type)	Miocene/Pliocene
Echitricolporites mcneillyi Germeraad et al	Asteroideae (Heliantheae, Ambrosiinae)	Miocene/Pliocene
Echitricolporites sp. *sensu* Wang [19]	Asteroideae	Miocene/Pliocene
Tubulifloridites sp. *sensu* Wang [19]	Asteroideae?	Miocene/Pliocene
Tricolporopollenites kozaniensis Weiland	Carduoideae?	Miocene/Pliocene
Tubulifloridites macroechinatus Nagy	Carduoideae?	Miocene/Pliocene
ricolporopollenites spinilophatus Trevisan	Cichorioideae (Cichorieae, *Sonchus type*)	Miocene/Pliocene
Cichorium type *sensu* Blackmore [4]	Cichorioideae (Cichorieae)	Miocene/Pliocene
Fenestrites longispinosus Lorente	Cichorioideae (Cichorieae)	Miocene/Pliocene
Liguliflorídites sp. *sensu* Couper [7]	Cichorioideae (Cichorieae)	Miocene/Pliocene
Sonchus type *sensu* Blackmore [4]	Cichorioideae (Cichorieae)	Miocene/Pliocene
Fenestrites spinosus Hammen	Cichoroideae (Cichorieae/Vernoniae)	Miocene/Pliocene
Artemisiaepollenites sellularis Nagy	Asteroideae (Anthemideae)	Miocene/Pliocene
Artemisiaepollenites leatus Zheng	Asteroideae (Anthemideae)	Pliocene
Scorzonera type *sensu* Blackmore [4]	Cichorioideae (Cichorieae)	Pliocene
Tubulifloridites pleistocenicus Martin	Asteroideae	Pliocene/Pleistocene

S4 Asteraceae genes

Table S2: Asteraceae genes Number of taxa (total and sampled) and genes used in phylogenetic analyses for each clade of Asteraceae.

Clades	Number of Species	Sampled Species	Age	Genes
Barnadesioideae	92	70	64.41	ITS, trnL, matK except 7 species of the backbone that include 12 markers (see Panero et al. 2015)
Famatinanthoideae	1	1	60.82	accD, atpB, matK, ndhD, ndhF, ndhI, ndhJKC, rbcL, rpoB, rpoC1exon2, trnL intron-trnL-F spacer
Mutisioideae	637	210	40.55	ITS, ndhF except 12 species of the backbone that include 12 markers
Stifftioideae	35	4	34.84	accD, atpB, matK, ndhD, ndhF, ndhI, ndhJKC, rbcL, rpoB, rpoC1exon1, rpoC1exon2, trnL intron-trnL-F spacer
Wunderlichioideae	48	6	48.88	accD, atpB, matK, ndhD, ndhF, ndhI, ndhJKC, rbcL, rpoB, rpoC1exon1, rpoC1exon2, trnL intron-trnL-F spacer
Gochnatioideae	85	32	42.60	ndhF, trnL except 3 species of the backbone that include 12 markers
Hecastocleidoideae	1	1	46.86	accD, atpB, matK, ndhD, ndhF, ndhI, ndhJKC, rbcL, rpoB, rpoC1exon1, rpoC1exon2, trnL intron-trnL-F spacer
Carduoideae	2865	373	24.26	psbA-trnH, matK except 5 species of the backbone that include 12 markers
Gymnarrhenoideae	2	1	38.19	accD, atpB, matK, ndhD, ndhF, ndhI, ndhJKC, rbcL, rpoB, rpoC1exon1, rpoC1exon2, trnL intron-trnL-F spacer
Corymbioideae	7	1	32.13	accD, atpB, matK, ndhD, ndhF, ndhI, ndhJKC, rbcL, rpoB, rpoC1exon1, rpoC1exon2, trnL intron-trnL-F spacer
Inuleae	680	105	20.03	psbA-trnH, trnL-F except 1 species of the backbone that include 12 markers
Helianthodae	6611	510	21.41	ITS, matK except 6 species of the backbone that include 12 markers
Senecionodae-Asterodae	10589	648	26.60	ITS, trnL-trnF except 3 species of the backbone that include 12 markers
Cichorioideae	3994	759	28.51	ITS, trnL-trnF except 4 species of the backbone that include 12 markers
Pertyoideae	70	2	17.35	accD, atpB, matK, ndhD, ndhF, ndhI, ndhJKC, rbcL, rpoB, rpoC1exon1, rpoC1exon2, trnL intron-trnL-F spacer
Total	25717	2723		

S5 Poaceae genes

Table S3: Poaceae genes Number of taxa (total and sampled) and genes used in phylogenetic analyses for each clade of Poaceae according to Spriggs et al (2014). Age estimations correspond to the calibration scenario 1 of Spriggs et al (2014)[17]

Clades	Number of Species	Sampled Species	Age	Genes
Pharoideae	12	1	57.80	matK, ndhF, rbcL
Puelioideae	11	1	53.98	matK, ndhF, rbcL
Ehrhartoideae	112	66	33.77	GPA1, ITS, matK, ndhC, ndhF, psbHpetB, psbZ, rbcL rps19, trnHpsbA, ycf3
Bambusoideae	1441	418	23.27	matK, ndhF, rbcL, ITS, psbA-trnH, rpL16, rpL32, rps16-trnQ, trnC-rpoB, trnL-trnF, trnTtrnD
Pooideae	3850	1335	38.25	DMC1, ITS, matK, ndhF, pgk1, psbAtrnH, rbcL, rpb2, rpoA, rps19, trnKrps16, trnLtrnF
Aristidoideae	365	125	27.95	ITS, matK, ndhF, rbcL, rpL16, trnL-trnF
Centotheceae	33	33	29.66	ndhF, rbcL, matk, trnLtrnF, phyB, ITS
Andropogoneae	1274	250	16.97	ITS, phyB, trnLtrnF, matK, ndhF, rbcL
Paspaleae	664	168	21.01	trnL-trnF, rbcL, psbA-trnH, atpB-rbcL, trnG, rpL16, ndhF, matK, ITS
Paniceae-Gynerieae	1254	387	21.29	ITS, kn1, matK, ndhF, rbcL, rpL16, trnLtrnF
Danthonioideae	281	234	28.71	ndhF, rbcL, atpB-rbcL, ITS, psbM, rpl16, rpoC2, trnC-trnD, trnL, ycf6-trnC, trnT-trnL, matK
Chloridoideae	1721	534	32.91	ITS, matK, ndhF, rbcL rl16 rps16, rps3, trnL-trnF
Arundinoideae-Micrairoideae	188+46	43	30.38	ITS, rpoC2, matK, ndhF, rbcL
Total	11256	3595		

S6 Environmental CO_2 curve

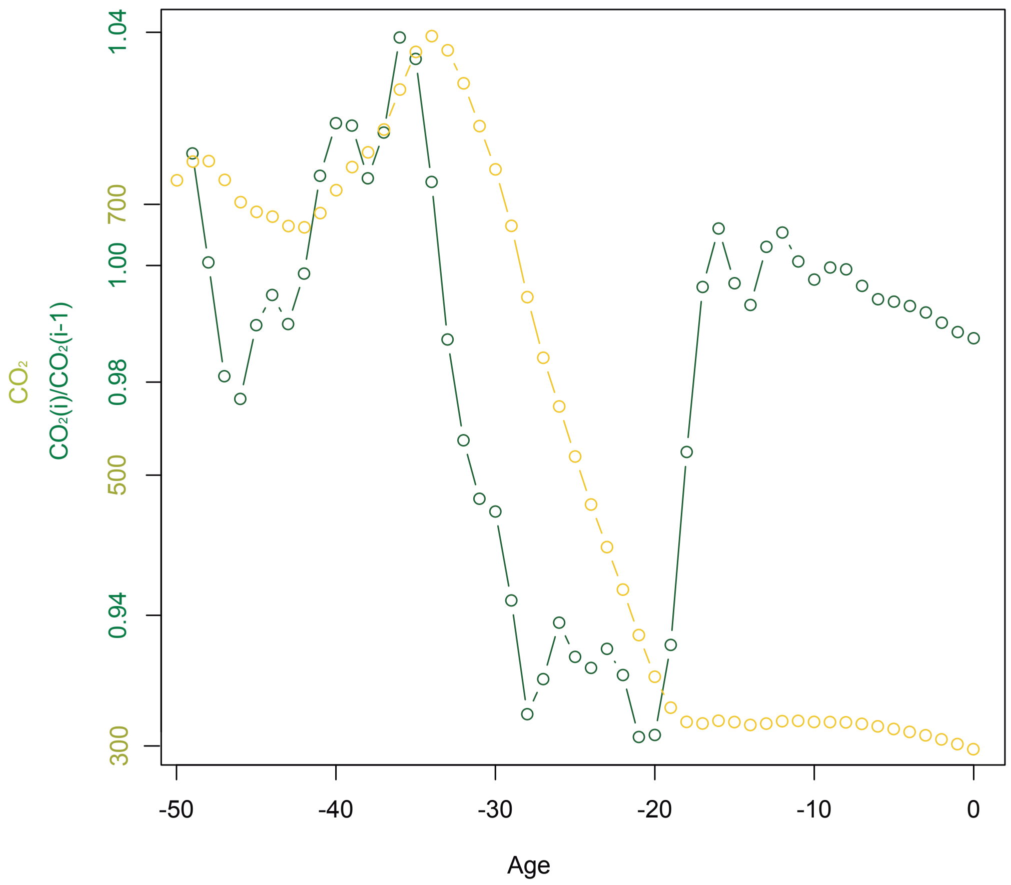

Figure S3: Environmental CO_2 and changes in CO_2 between time intervals. The lighter green line shows the environmental CO_2 as was used in the environmentally-dependent diversification rates models. The darker green line shows the changes in CO_2 over time, which might translate into changes in diversification rates if diversification rates are correlated with environmental CO_2.

S7 Models and Priors for the environmental correlation analyses

S7.1 Fixed

Our first model, which is equivalent to the exponential environmentally-dependent diversification model of Condamine et al. [6], has two parameters per speciation and extinction rate; the initial rate λ_0 and μ_0 and the correlation factor β, with

$$\lambda_0 \sim \text{Uniform}(0, 100) \tag{S1}$$
$$\ln(\lambda_i) = \ln(\lambda_{i-1}) + \beta_\lambda \times \Delta\text{CO}_2 \quad . \tag{S2}$$

Table S4: Model parameter names and prior distributions for the fixed model.

Parameter	X	$f(X)$
Speciation at present	λ_0	Uniform(0,100)
Extinction at present	μ_0	Uniform(0,100)
Speciation Correlation	β_λ	Normal(0,1)
Extinction Correlation	β_μ	Normal(0,1)

S7.2 UC

The second models extends our first model by additional "errors", or variation, on top of the diversification rate variation due to the environmental variable. Thus, we additionally have a variance parameter σ^2 as well as ϵ_λ and ϵ_μ per interval. The resulting speciation and extinction rates are computed by

$$\lambda_0 \sim \text{Uniform}(0, 100) \tag{S3}$$
$$\ln(\hat{\lambda}_i) = \ln(\hat{\lambda}_{i-1}) + \beta_\lambda \times \Delta\text{CO}_2 \tag{S4}$$
$$\sigma \sim \text{halfCauchy}(0, 1) \tag{S5}$$
$$\epsilon_i \sim \text{Normal}(0, \sigma) \tag{S6}$$
$$\ln(\lambda_i) = \ln(\hat{\lambda}_i) + \epsilon_i \quad . \tag{S7}$$

Table S5: Model parameter names and prior distributions for the UC model.

Parameter	X	$f(X)$
Speciation at present	λ_0	Uniform(0,100)
Extinction at present	μ_0	Uniform(0,100)
Speciation Variation	σ_λ	HalfCauchy(0,1)
Extinction Variation	σ_μ	HalfCauchy(0,1)
Speciation Correlation	β_λ	Normal(0,1)
Extinction Correlation	β_μ	Normal(0,1)
Per interval log-speciation rate variation	ϵ_λ	Normal(0,σ_λ)
Per interval log-extinction rate variation	ϵ_μ	Normal(0,σ_μ)

S7.3 Gaussian Markov Random Field (GMRF)

The GMRF model has autocorrelated rate variation compared to the uncorrelated variation from the UC model. The speciation and extinction rates are computed by

$$\begin{aligned}
\lambda_0 &\sim \text{Uniform}(0,100) &\text{(S8)}\\
\sigma &\sim \text{halfCauchy}(0,1) &\text{(S9)}\\
\epsilon_i &\sim \text{Normal}(0,\sigma) &\text{(S10)}\\
\ln(\lambda_i) &= \ln(\lambda_{i-1}) + \beta_\lambda \times \Delta\text{CO}_2 + \epsilon_i \quad . &\text{(S11)}
\end{aligned}$$

We additionally used a global scaling parameter for the variation of diversification of $\zeta = 0.587405 \times N$, where N is the number of epochs, so that we expect one order of magnitude variation over the course of the age of the phylogeny [15]. Alternatively, the parameter ζ could be place into the prior distribution on σ by using a halfCauchy$(0,\zeta)$ prior distribution instead. Both approaches are equivalent.

Table S6: Model parameter names and prior distributions for the GMRF model.

Parameter	X	$f(X)$
Speciation at present	λ_0	Uniform(0,100)
Extinction at present	μ_0	Uniform(0,100)
Speciation Variation	γ_λ	HalfCauchy(0,1)
Extinction Variation	γ_μ	HalfCauchy(0,1)
Speciation Correlation	β_λ	Normal(0,1)
Extinction Correlation	β_μ	Normal(0,1)
Per interval log-speciation rate difference	$\ln(\Delta_\lambda)$	Normal(0,$\gamma_\lambda\zeta$)
Per interval log-extinction rate difference	$\ln(\Delta_\mu)$	Normal(0,$\gamma_\mu\zeta$)

S7.4 HSMRF

The HSMRF model allows for interval specific variance parameters γ_i^2. This allows for some epochs to be more variable, while most epochs will actually be less variable [15]. The resulting speciation and extinction rates are thus computed by

$$\begin{aligned}
\lambda_0 &\sim \text{Uniform}(0,100) &\text{(S12)}\\
\sigma &\sim \text{halfCauchy}(0,1) &\text{(S13)}\\
\gamma_i &\sim \text{halfCauchy}(0,1) &\text{(S14)}\\
\epsilon_i &\sim \text{Normal}(0,\sigma\gamma_i\zeta) &\text{(S15)}\\
\ln(\lambda_i) &= \ln(\lambda_{i-1}) + \beta_\lambda \times \Delta\text{CO}_2 + \epsilon_i \quad . &\text{(S16)}
\end{aligned}$$

As before, we additionally used a global scaling parameter for the variation of diversification of $\zeta = 0.587405 \times N$ so that we expect one order of magnitude variation over the course of the age of the phylogeny [15].

Table S7: Model parameter names and prior distributions for the HSMRF model.

Parameter	X	$f(X)$
Speciation at present	λ_0	Uniform(0,100)
Extinction at present	μ_0	Uniform(0,100)
Speciation Variation	σ_λ	HalfCauchy(0,1)
Extinction Variation	σ_μ	HalfCauchy(0,1)
Speciation Correlation	β_λ	Normal(0,1)
Extinction Correlation	β_μ	Normal(0,1)
Per interval speciation rate variation factor	$\gamma_{\lambda,i}$	HalfCauchy(0,1)
Per interval extinction rate variation factor	$\gamma_{\mu,i}$	HalfCauchy(0,1)
Per interval log-speciation rate difference	$\ln(\Delta_{\lambda,i})$	Normal(0,$\gamma_{\lambda,i}\sigma_\lambda\zeta$)
Per interval log-extinction rate difference	$\ln(\Delta_{\mu,i})$	Normal(0,$\gamma_{\mu,i}\sigma_\mu\zeta$)

S8 Diversification rates with phylogenies

S8.1 Diversification shifts against simplified phylogenies

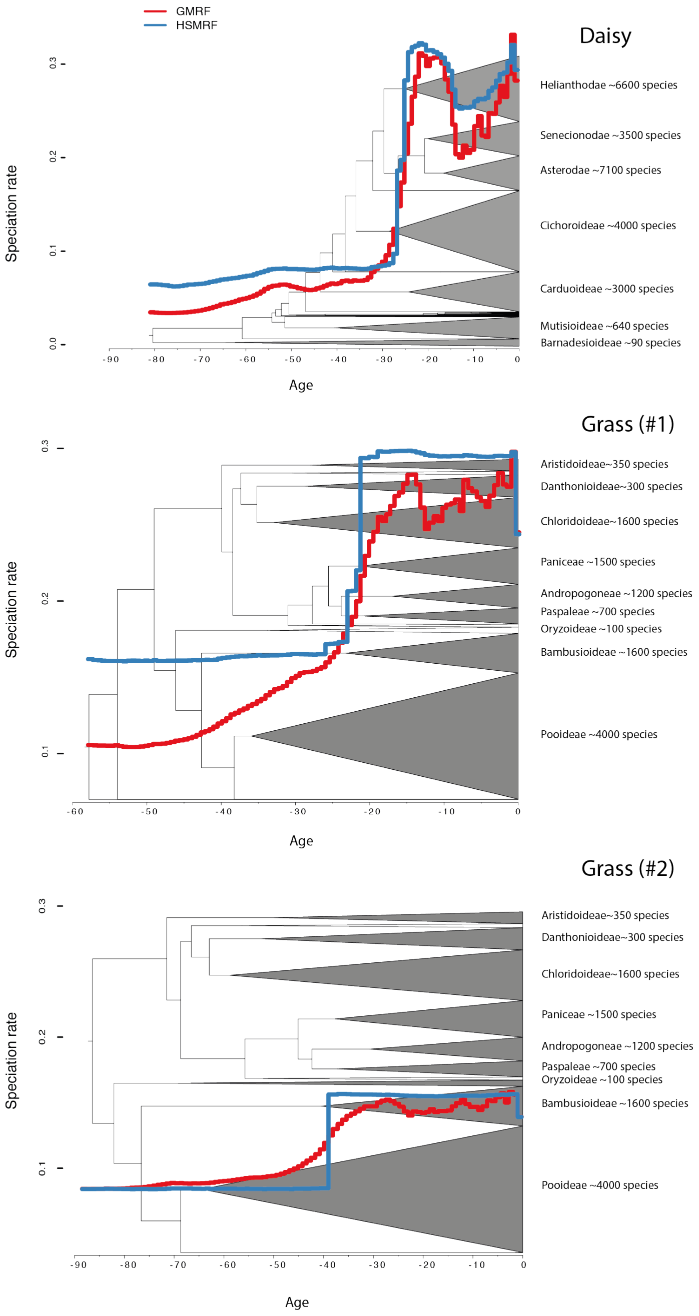

Figure S4: Speciation rates using the GMRF (red) and HSRMF (blue) models with daisy and grass (#1 and #2) phylogenies. Note that the most pronounced increase in speciation rates in both families occurred during the divergence of the largest clades and when all major subfamilies were already present. This indicates the global (i.e., tree-wide) impact of diversification rate change.

S8.2 Diversification shifts among lineages

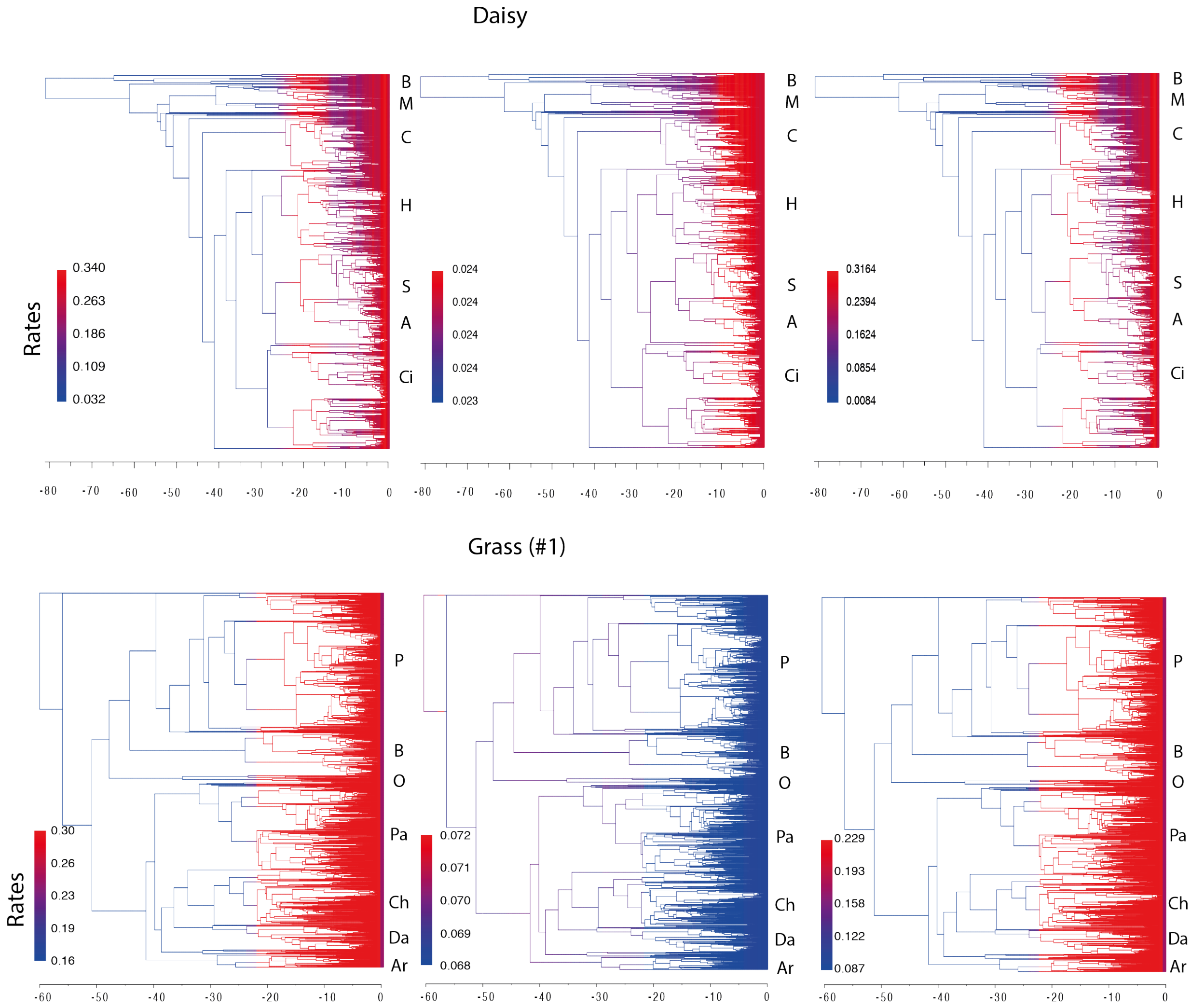

Figure S5: Speciation (left), extinction (middle) and diversification (right) rates using the best fitting models (GMRF for daisies and HSRMF model for grasses (#1)). For daisies, B: Barnadesioideae; M: Mutisioideae; C: Carduoideae; H: Helianthodae; S:Senecionodae; A:Asterodae; Ci:Cichorioideae. For grasses, P:Pooideae; B:Bambusoideae; O:Oryzoideae; P:Paniceae; Ch:Chlorioideae; Da:Danthonioideae; Ar:Aristidoideae. Note that the most pronounced increase in diversification rates occurred during the last ∼30 Mya in both families. Also note that diversification rates change while all subfamilies diversified. This shows the global (i.e., tree-wide) impact of the diversification rate change compared to a single or few clades driving the diversification rate changes.

S9 The effect of different prior models on diversification rates

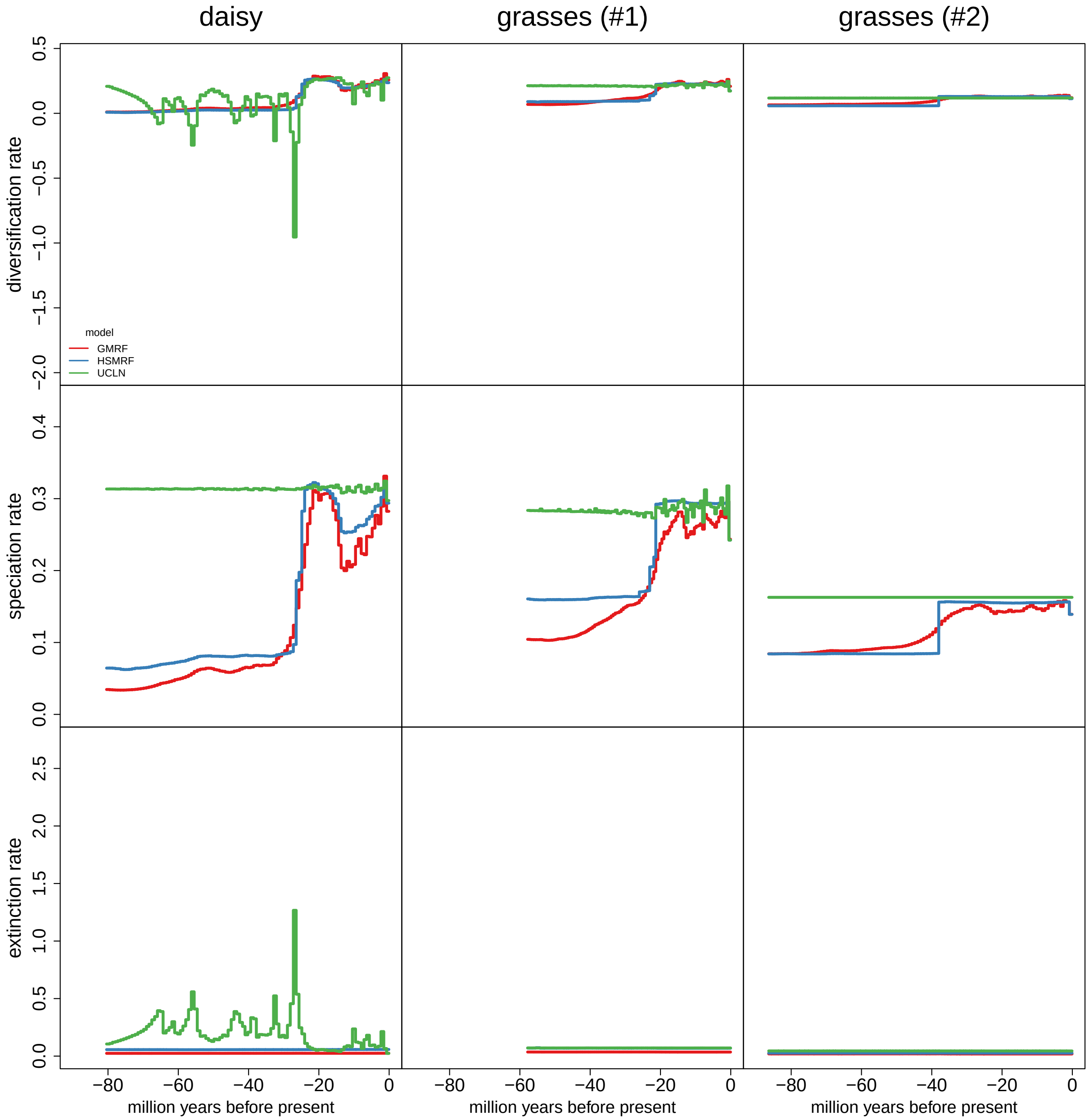

Figure S6: Diversification rate estimates using different prior models. The speciation, extinction and net-diversification rate estimates using the GMRF (red), HSRMF (blue) and UCLN (green) prior models. The rates were estimated using 100 epochs (for other numbers of epochs see Figure S7). The two autocorrelated models qualitatively agree on the estimated rates and infer diversification rate shifts driven by changes in the speciation rate. As expected, the GMRF model produces smoother rates while the HSMRF model produces more discrete jumps. The UCLN model produces strong variation which is a clear result of over-fitting (see Figure S8). Also note that this results cannot be due to non-identifiability of diversification rates because the episodic birth-death model used here is identifiable [13].

S10 The effect of number of epochs on diversification rates

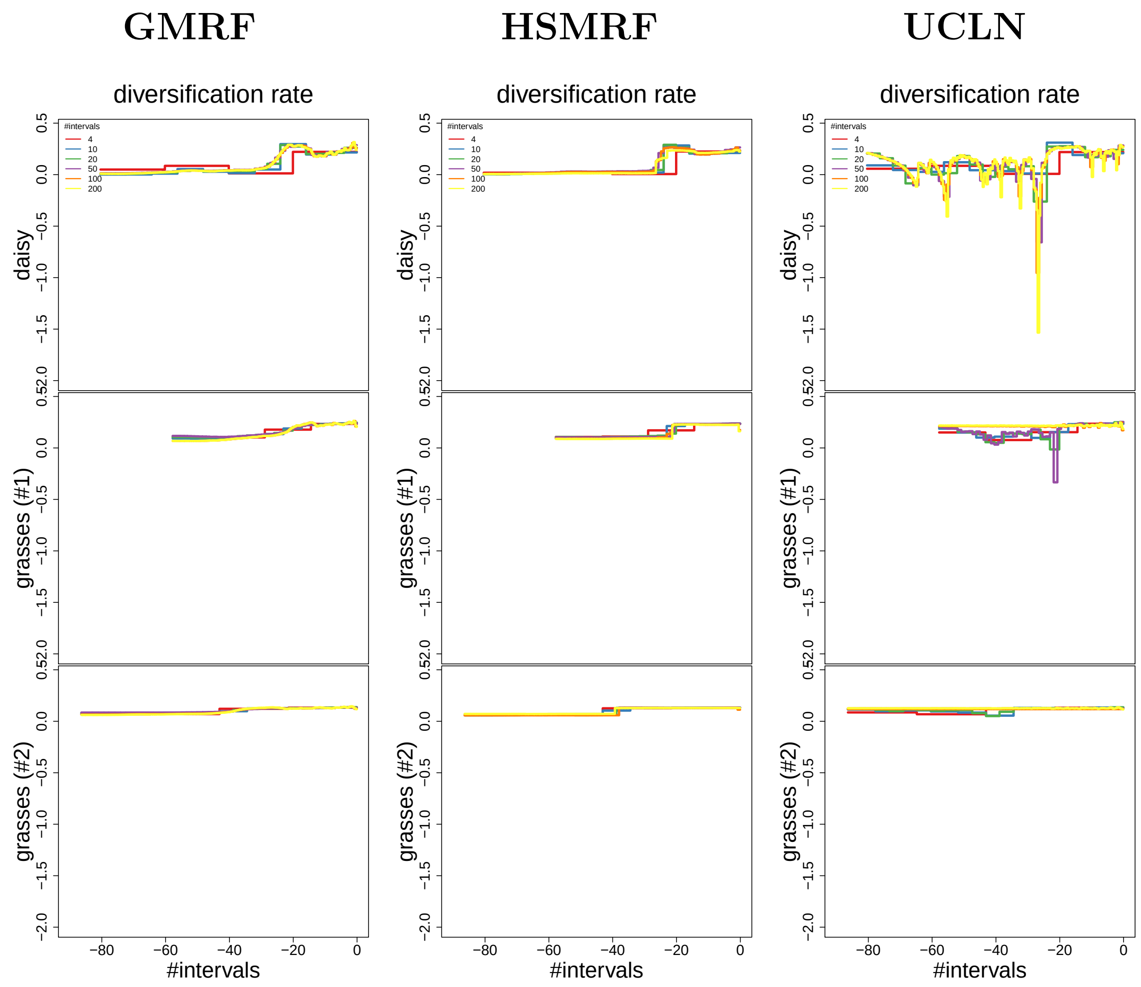

Figure S7: Diversification rate estimates for different number of epochs. We estimated diversification rates using $N = \{4, 10, 20, 50, 100, 200\}$ epochs for all three diversification prior models (GMRF, HSMRF and UCLN). The two autocorrelated models estimate smoother rate functions with higher number of intervals, as previously observed by Magee et al[15]. The uncorrelated model over-fitted when many intervals where used (see Figure S8).

S11 Model selection of diversification models

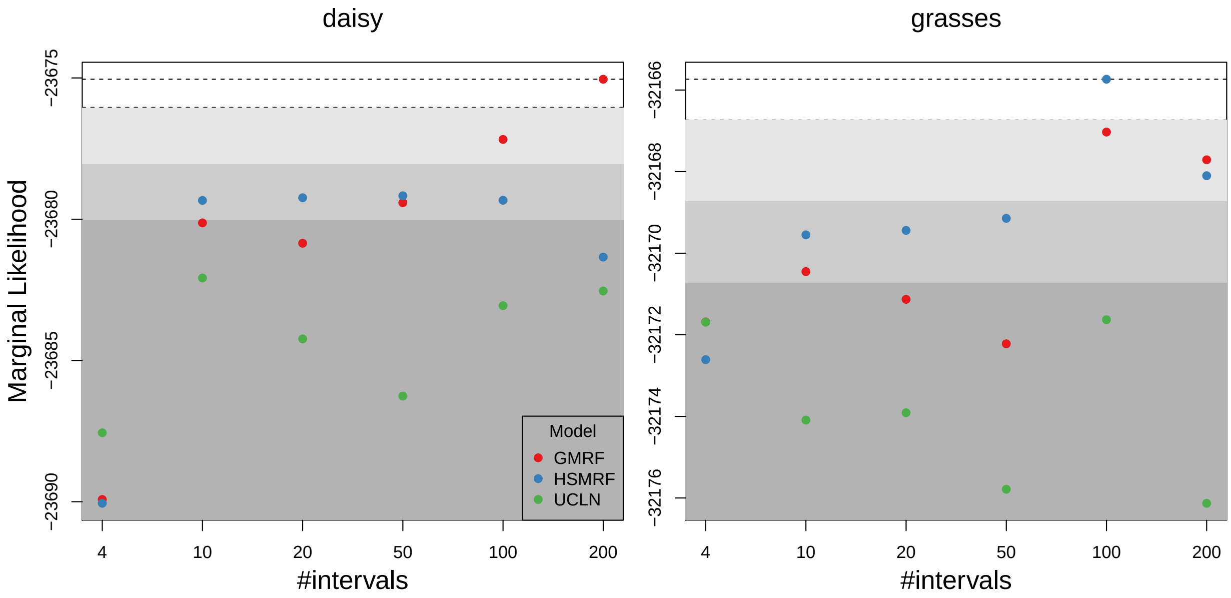

Figure S8: Model selection between the three diversification rate prior models and for different number of epochs. We estimated marginal likelihoods for all three diversification prior models (GMRF, HSMRF and UCLN) and for varying number of epochs, $N = \{4, 10, 20, 50, 100, 200\}$. The colored areas show (i) no significant support in white, (ii) support in light gray, (iii) strong support in gray, and (iv) decisive support in dark gray according to the common Bayes factor thresholds [12]. The UCLN model was always decisively rejected. The best fitting model for the daisy phylogeny was the GMRF with 200 epochs and the HSRMF with 100 epochs for the grasses phylogeny. Interestingly, only for $N = 4$ epochs was the UCLN model better (daisy dataset) or equially good (grasses dataset) but more epochs resulted in worse model fit (best fit for daisies was 10 epochs and 4 epochs for grasses).

S12 The effect of different taxon sampling methods

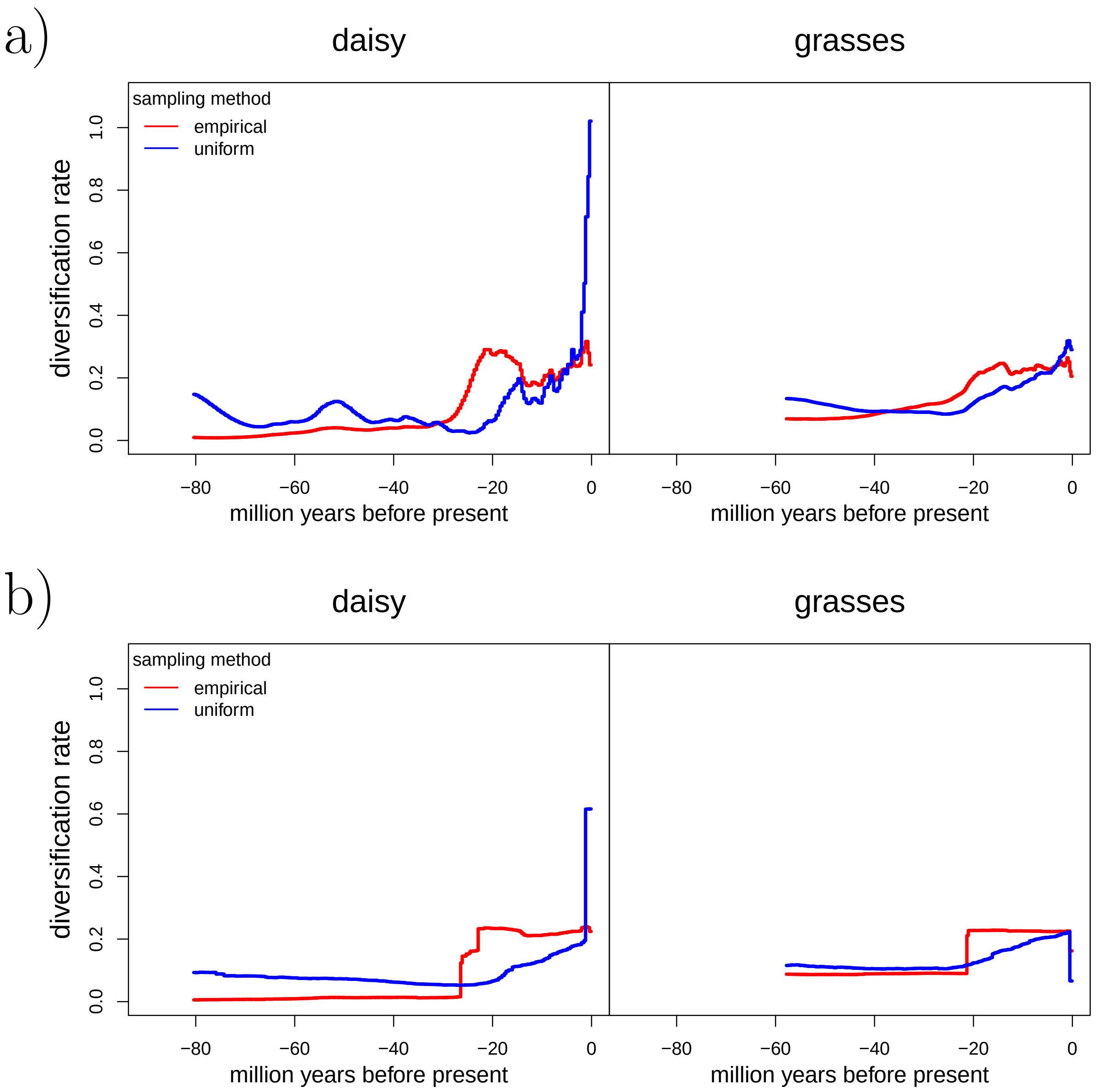

Figure S9: Diversification rate estimates using different incomplete taxon sampling methods. We estimated the diversification rates using our newly developed *empirical* taxon sampling and the previously developed *uniform* taxon sampling. The diversification rates were estimates using a) the GMRF diversification rate prior model and b) the HSRMF diversification rate prior model. For each model with assumed $N = 200$ epochs. The estimated diversification rate differ depending on the incomplete taxon sampling method, but are similar between diversification rate prior models (see Figure S6). Thus, using the correct incomplete taxon sampling approach is crucial for unbiased estimation of diversification rates.

S13 The effect of different models on correlations to the environmental variable

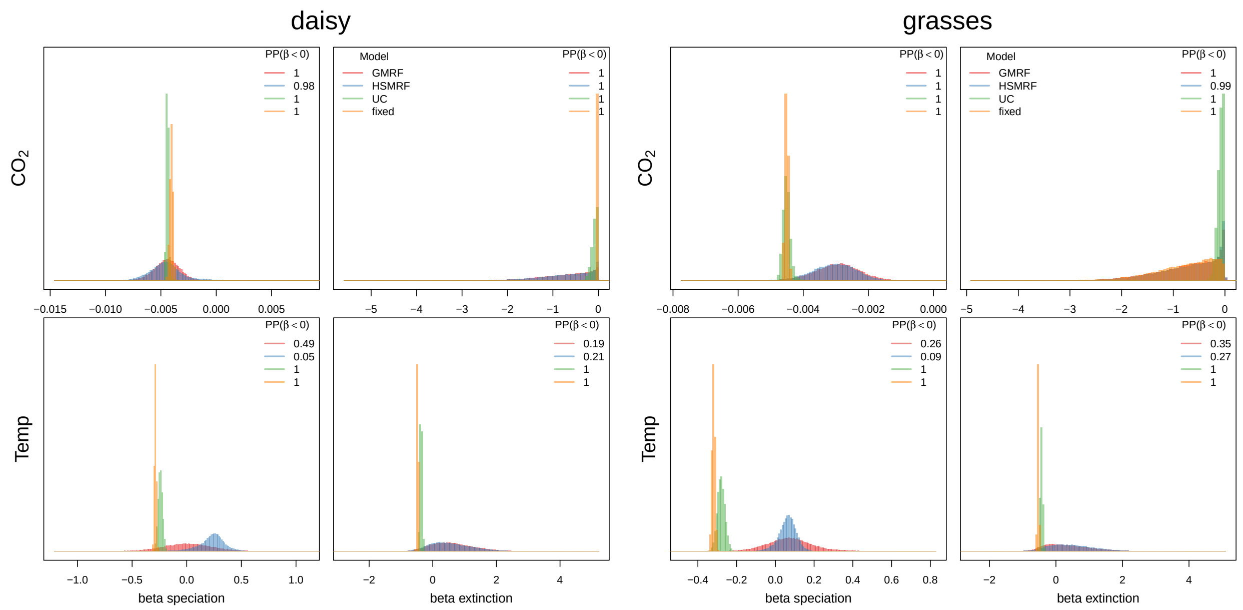

Figure S10: Estimating correlation between diversification rates and two environmental variables. We estimated the correlation between atmospheric CO_2 and paleo-temperature (with environmental variables averaged in 1MY bins) to speciation and extinction rates using our four environmentally-dependent diversification models: *fixed*, UC, GMRF and HSMRF. The inset posterior probabilities show the posterior probability that the correlation factor β is smaller than 0 (i.e., a negative correlation). If the posterior probability is close to one, then there is a significant negative correlation and if the posterior probability is close to zero, then there is a significant positive correlation. The speciation rate is negatively correlated with atmospheric CO_2 for the both the daisy and grasses datasets regardless of the chosen model. The extinction rate is also negatively correlated and shows the same trend as the speciation rate. The correlation between paleo-temperature and speciation rate is less certain: the *fixed* and UC model infer a negative correlation whereas the GMRF and HSMRF infer no correlation or a positive correlation. The HSMRF model was the best fit model for the paleo-temperature analyses (see Figure S11). The same trend is seen in both datasets for the daisies and grasses.

S14 Model selection of environmental variable correlated models

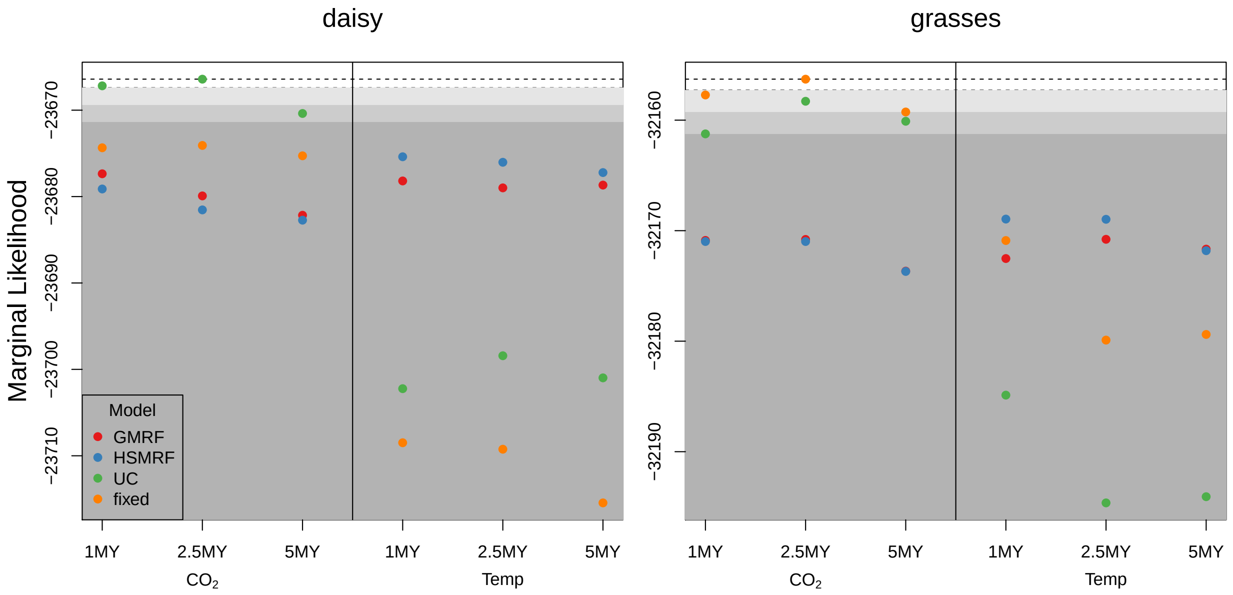

Figure S11: Marginal likelihood estimates for the four environmentally-dependent diversification models. We estimated marginal likelihoods using stepping stone sampling for the four environmentally-dependent diversification models (*fixed*, UC, GRMF and HSMRF) and two environmental variables (atmospheric CO_2 and paleo-temperature). Additionally, we binned the environmental variable in 1, 2.5 and 5 million year bins. We computed the marginal likelihoods for both the daisy and grasses dataset (calibration method #1). The shaded boxes represent significance levels according to standard Bayes factors[12]: slightly support (white), supported (light gray), strongly supported (dark gray) and decisively supported (dark gray). We found that for the daisy dataset the best predictor is atmospheric CO_2 with 2.5 MY bins using the UC model. The binning size did not impact the results and the order of preferred models remained unchanged. For the grasses dataset, the *fixed* model with atmospheric CO_2 as predictor variable in 2.5 MY bins received highest support. Interestingly, for the paleo-temperature as predictor variable the results flip with the two autocorrelated models (GMRF and HSMRF) receiving higher marginal likelihood than the *fixed* and UC model. This is particularly important as for the atmospheric CO_2 all models agreed on the negative correlation between CO_2 and speciation rates, whereas for the paleo-temperature the results qualitatively differed from negative correlation (*fixed* and UC) to no correlation or slighly positive correlation (GMRF and HSMRF); see Figure S10.

S15 The effect of binning on environmental variable correlated models

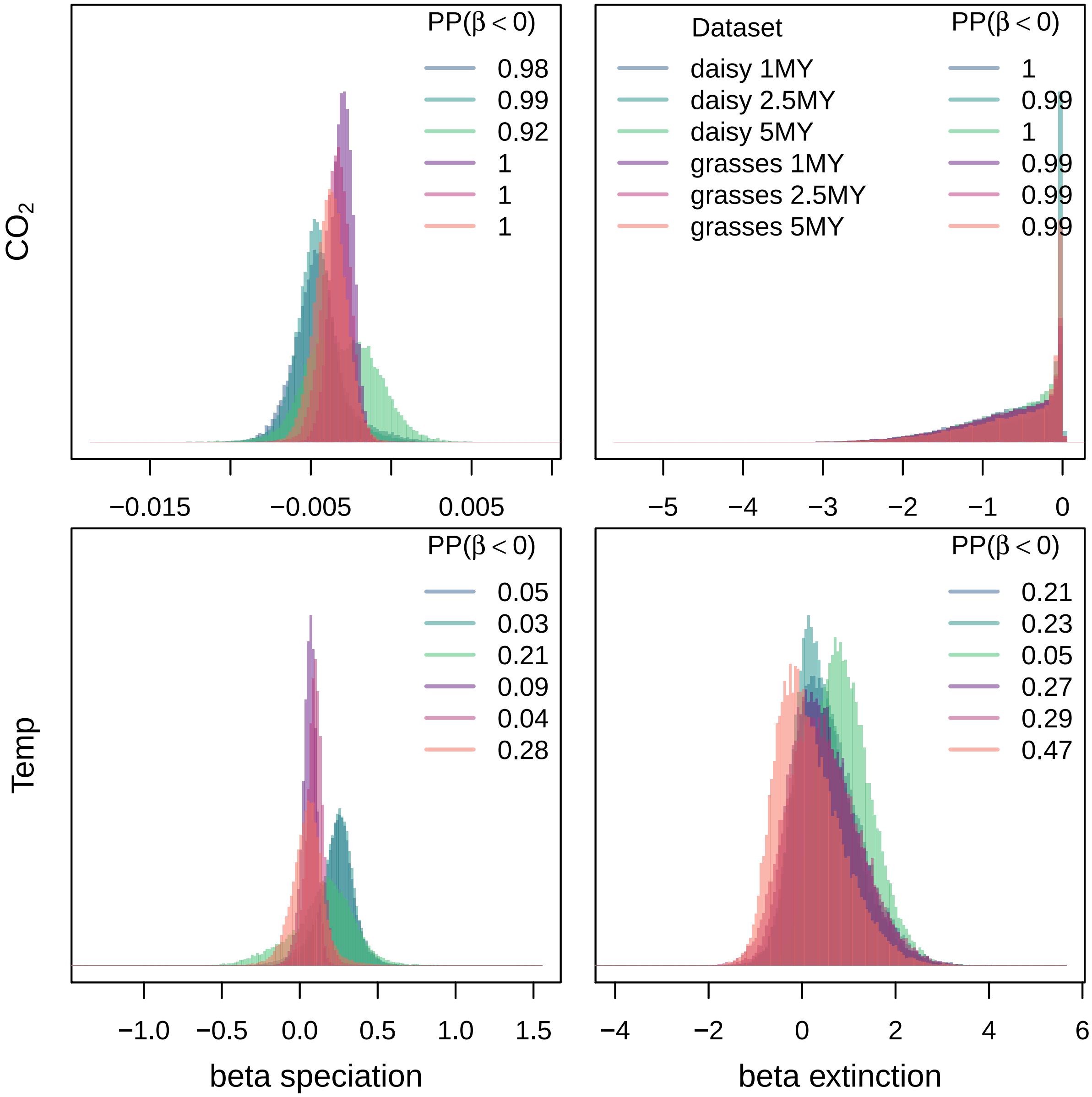

Figure S12: Estimated correlation factor for different bin size of the environmental variable. We estimated the correlation factor β between the speciation and extinction rates and the environmental variable (atmospheric CO_2 and paloe-temperature) using the HSRMF model. The environmental variables were computed as averages for bins of size 1, 2.5 and 5 MY. The inset posterior probabilities show the posterior probability that the correlation factor β is smaller than 0 (i.e., a negative correlation). If the posterior probability is close to one, then there is a significant negative correlation and if the posterior probability is close to zero, then there is a significant positive correlation. There is little effect on the correlation factor depending on the bin size, although a bin size of 5MY has the widest posterior distribution and is slightly shifted closer towards zero values and the posterior probabilities was less significant.

S16 The effect of node calibration on environmental variable correlated models

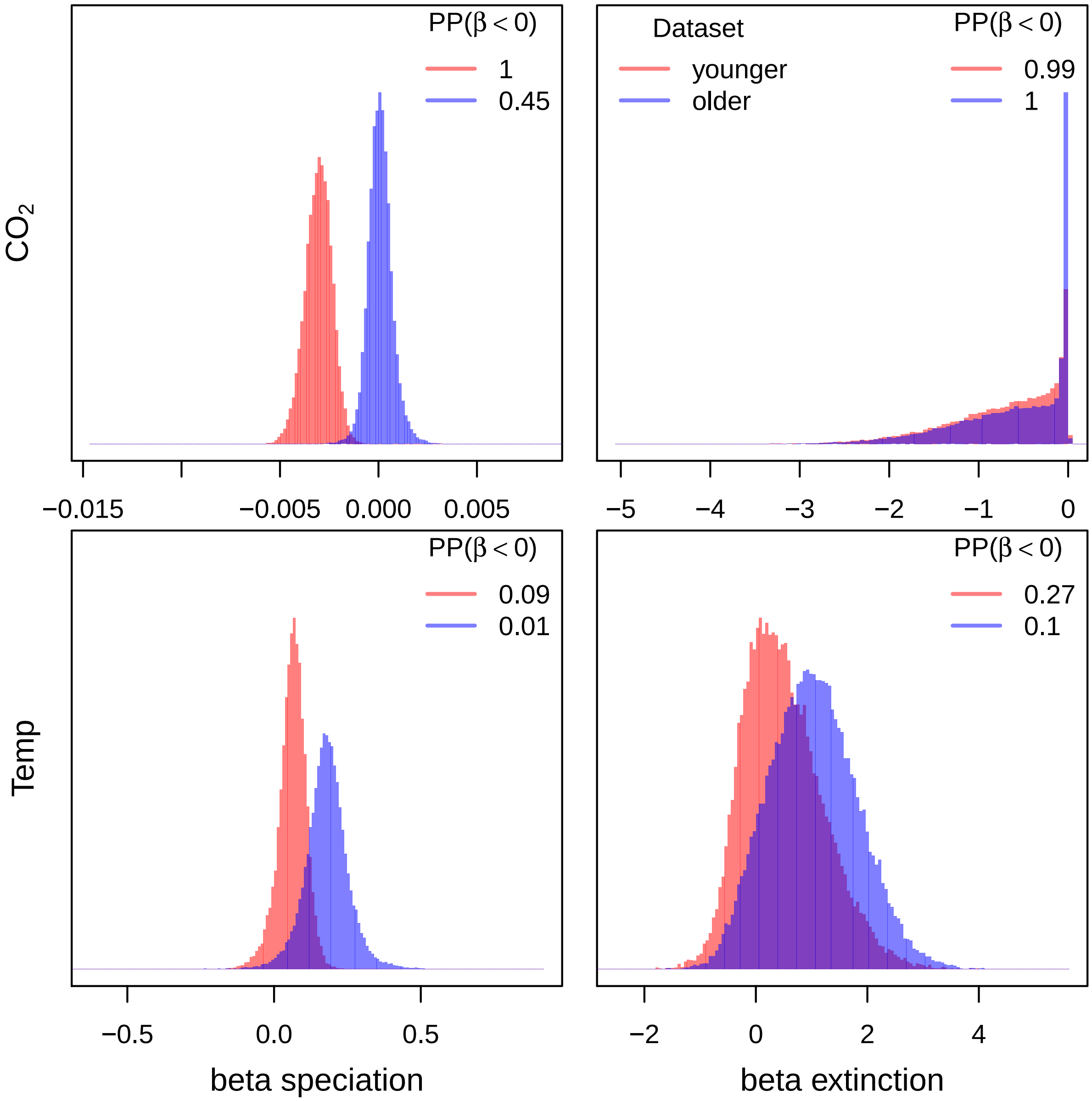

Figure S13: Estimated correlation factor β for the grasses dataset depending on node calibration scenario. We compared the correlation factor between speciation and extinction rates and environmental variables (atmospheric CO_2 and paleo-temperature with averages over 1MY bins). The inset posterior probabilities show the posterior probability that the correlation factor β is smaller than 0 (i.e., a negative correlation). If the posterior probability is close to one, then there is a significant negative correlation and if the posterior probability is close to zero, then there is a significant positive correlation. We applied the HSRMF model to the two grasses phylogenies obtained under different calibration scenarios (scenario #1 younger and scenario #2 older). For scenario #1 we estimate a negative correlation between atmospheric CO_2 and speciation rates but not correlation to paleo-temperature. For scenario #2 we estimate a positive correlation between speciation rates and paleo-temperature but no correlation to atmospheric CO_2.

S17 The effect of environmentally-dependent diversification models on diversification rates

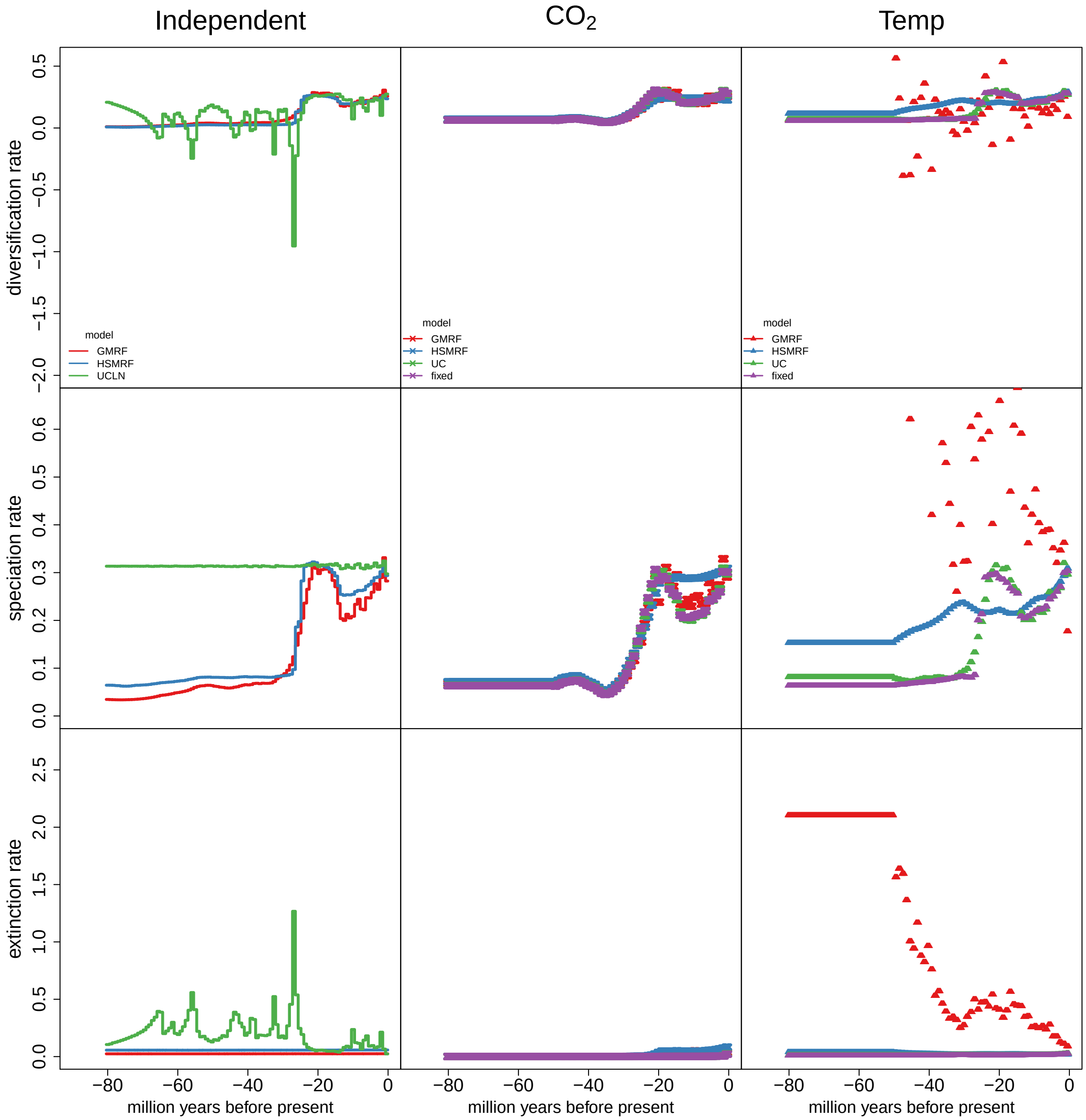

Figure S14: Estimated diversification rates for the daisy phylogeny. Here we compare the estimated diversification rates of our environmentally-dependent models to the null-hypothesis without environmental dependence. The environmentally dependent HSMRF and GMRF models yield extremely similar diversification rates compared with the environmentally independent diversification rate estimates. This shows that the diversification rates are not forced due the environmental correlation, but instead are driven by the information in the data (the phylogeny with divergence times). The uncorrelated (UC) rate models shows very different diversification rates for the correlation with paleo-temperature which confirms the disagreement in correlation factors (Figure S10). Unsurprisingly, the *fixed* environmentally-dependent diversification rate model show diversification rate closely following the environmental variable because no other source of rate-variation is allowed. Thus, all diversification rate variation in the *fixed* model, e.g., when using paleo-temperature as the environmental variable, is misleading because it is wrongfully enforced by the environmental variable.

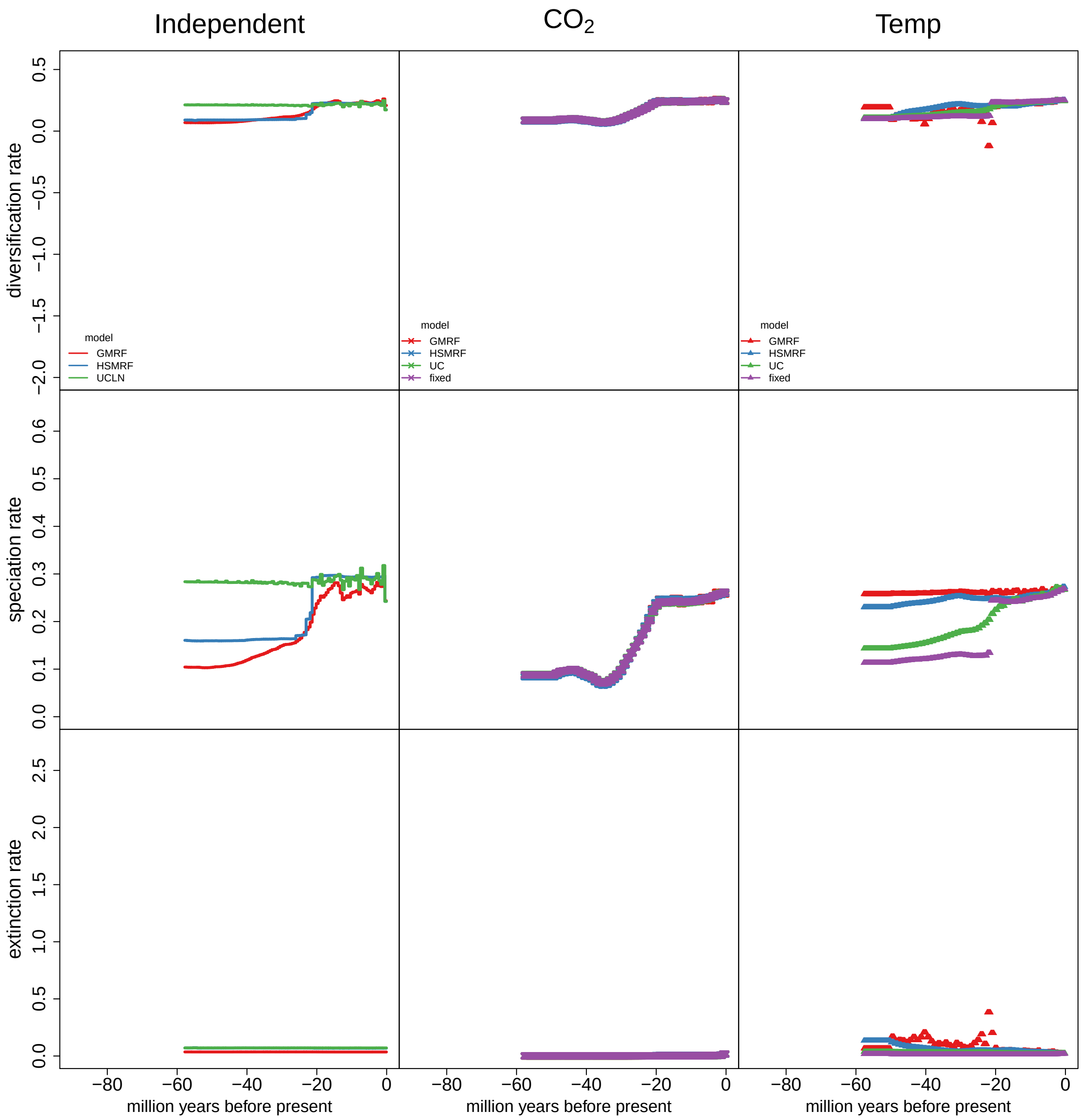

Figure S15: Estimated diversification rates for the grasses phylogeny (calibration scenario 1). Here we compare the estimated diversification rates of our environmentally-dependent models to the null-hypothesis without environmental dependence. For the CO_2 as the environmental driver, we see that all four environmentally-dependent diversification models provide very similar diversification rates. This confirms the agree that all models infer a correlation between CO_2 and diversification rates in grasses (Figure S10). Conversely, the diversification rates of all four models disagree if paleo-temperature is used. Thus, the correlation between paleo-temperature and diversification rates is unreliable and dependent on the specific model.

S18 Simulation study of environmentally-dependent diversification rates

S18.1 Simulated diversification rates under environmentally-dependent diversification rates models

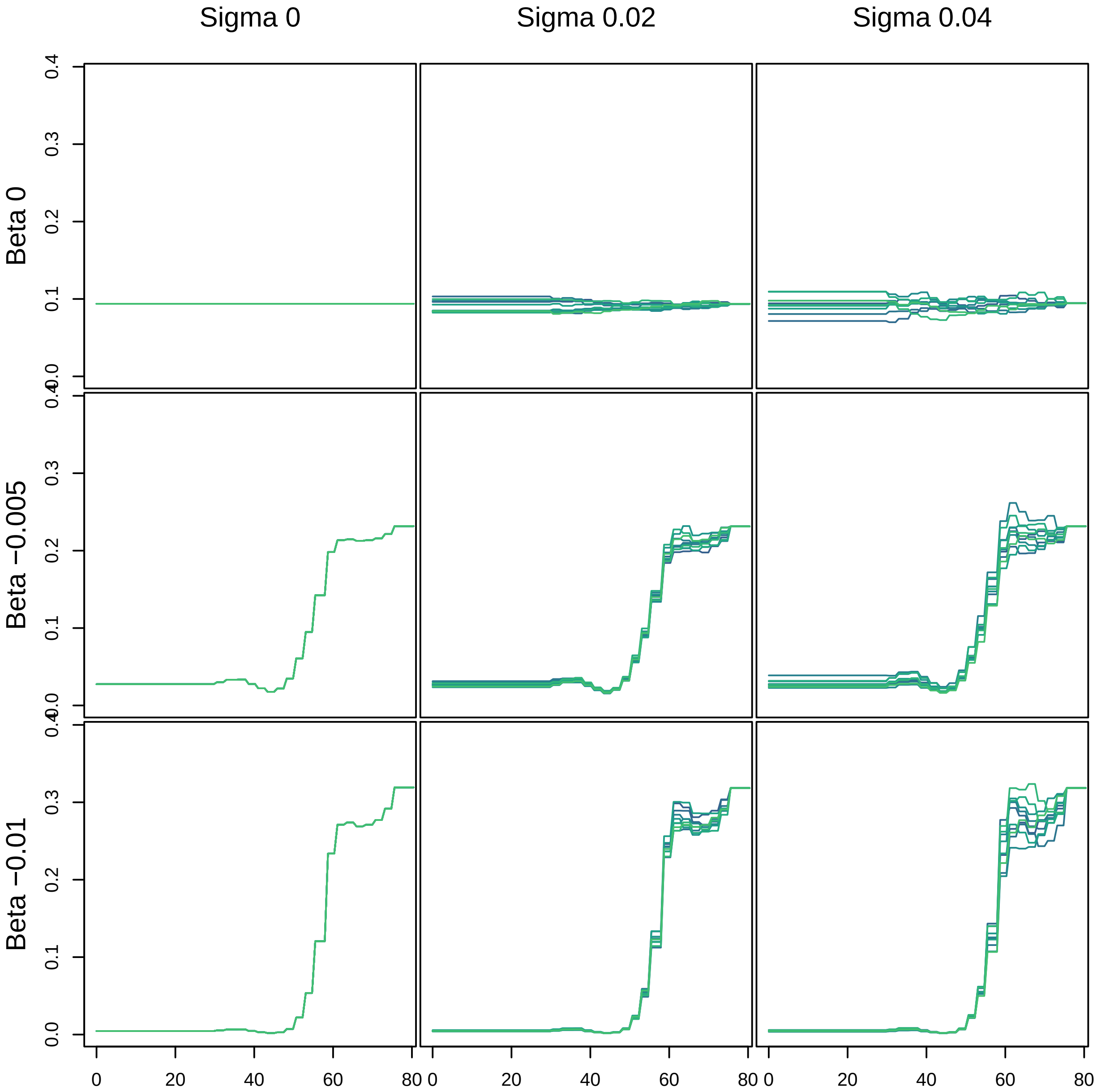

Figure S16: Speciation rated simulated under an environmentally-dependent GRMF diversification model. We simulated speciation rates with different correlation factors $\beta = \{0, -0.005, -.01\}$ and standard deviations $\sigma = \{0, 0.02, 0.04\}$. We used the atmospheric CO_2 as the environmental variable. When $\beta = 0$ in the simulations, then this model was equivalent to an environmentally-independent diversification model. When $\sigma = 0$ in the simulations, then this model was equal to the *fixed* environmentally-diversification model. When both $\beta = 0$ and $\sigma = 0$, then we simulated under a constant rate process (top left). This establishes the false positive rate in our simulations. Note that the autocorrelated model (GMRF) has overall more variation in speciation rates (e.g., top right) compared to the uncorrelated model (Figure S17) because the variation is additive over epochs in the autocorrelated model.

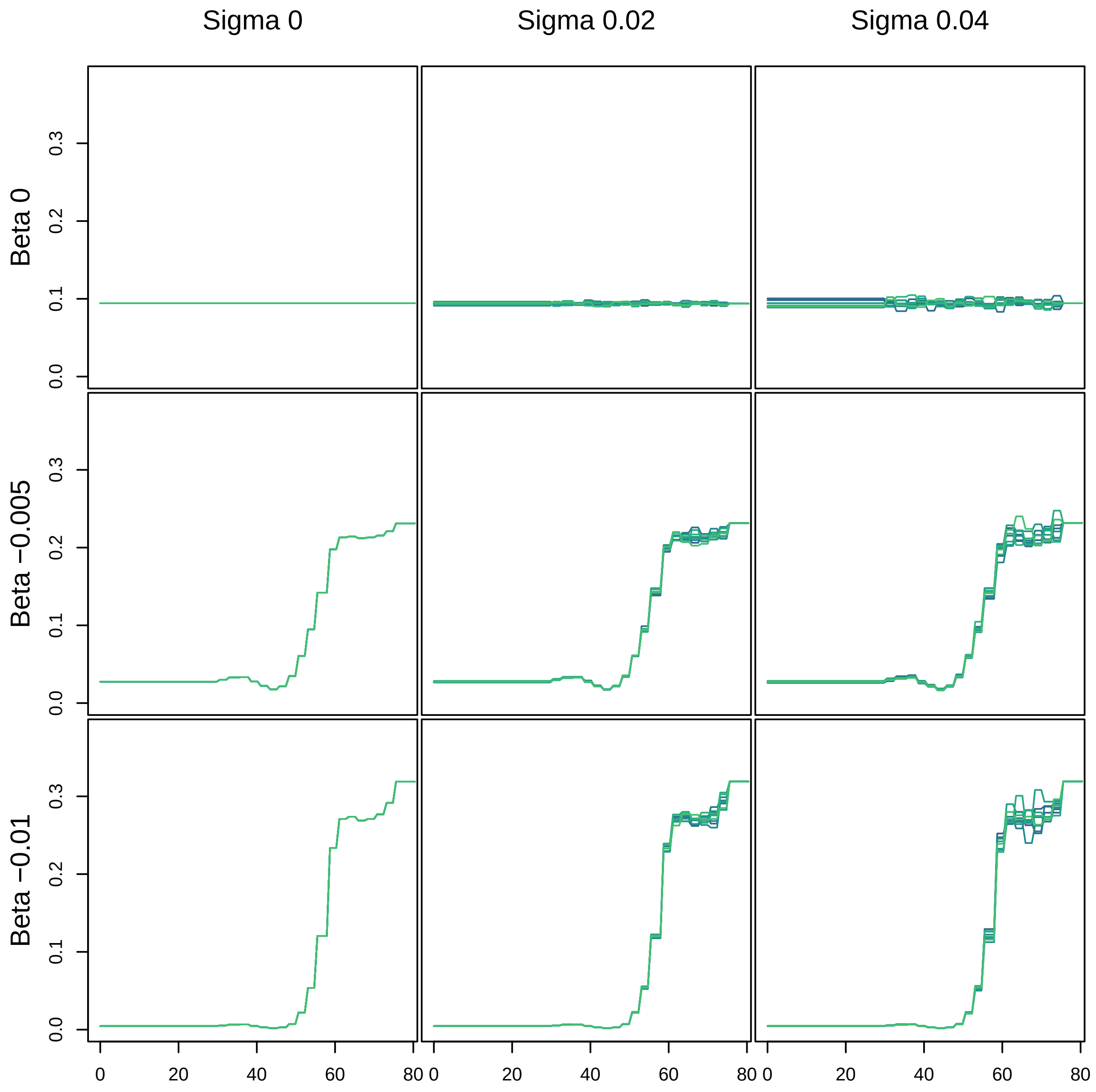

Figure S17: Speciation rated simulated under an environmentally-dependent UC diversification model. We simulated speciation rates with different correlation factors $\beta = \{0, -0.005, -.01\}$ and standard deviations $\sigma = \{0, 0.02, 0.04\}$. We used the atmospheric CO_2 as the environmental variable. When $\beta = 0$ in the simulations, then this model was equivalent to an environmentally-independent diversification model. When $\sigma = 0$ in the simulations, then this model was equal to the *fixed* environmentally-diversification model. When both $\beta = 0$ and $\sigma = 0$, then we simulated under a constant rate process (top left). This establishes the false positive rate in our simulations. Note that the autocorrelated model (GMRF, Figure S16) has overall more variation in speciation rates (e.g., top right) compared to the uncorrelated model because the variation is additive over epochs in the autocorrelated model.

S18.2 Simulated phylogenies under environmentally-dependent diversification rates

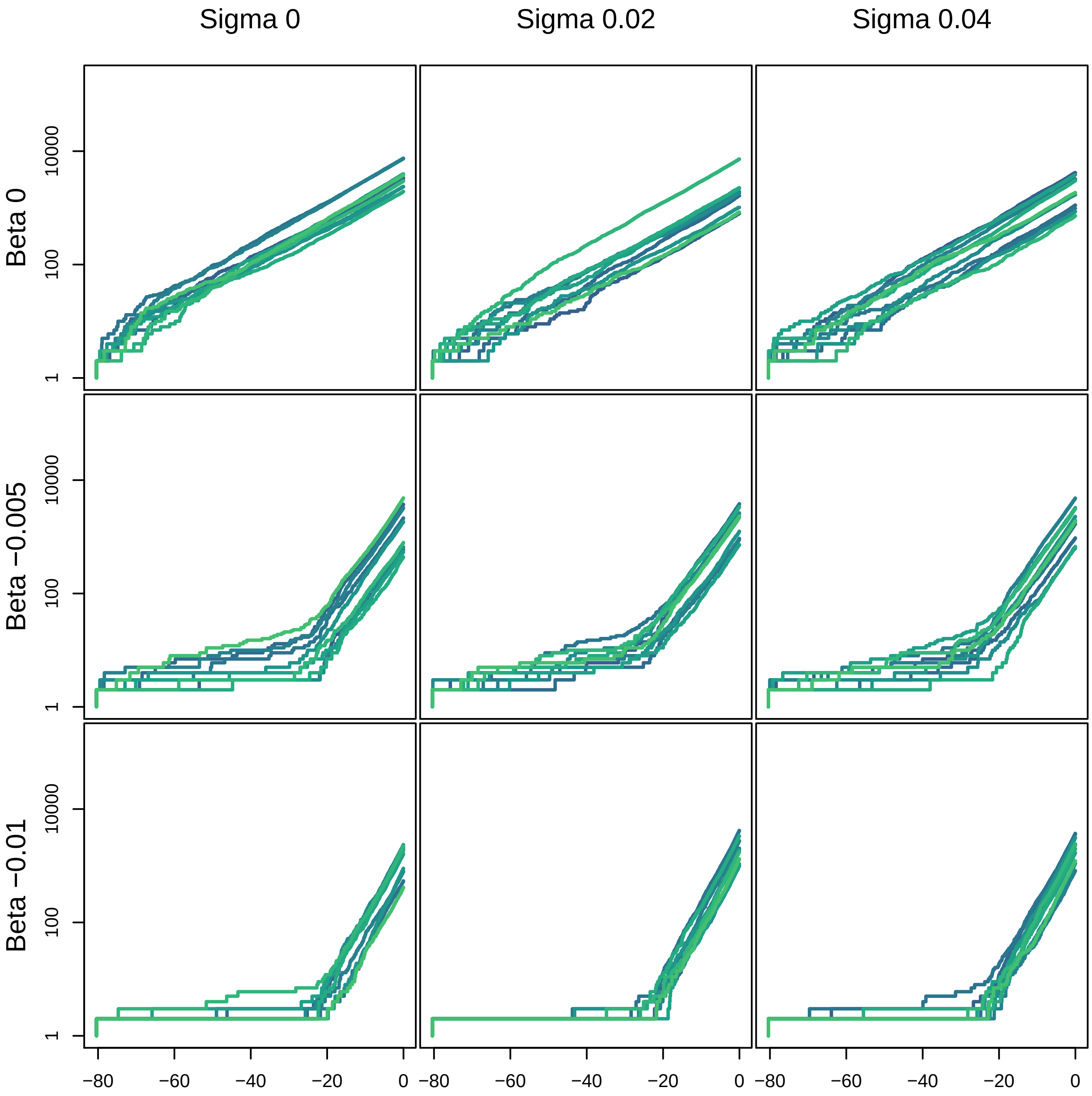

Figure S18: Lineage-through-time (LTT) curves of 10 phylogenies simulated under an environmentally-dependent GRMF diversification model. We used the simulated diversification rate trajectories shown and described in Figure S16. We nicely observe the log-linear LTT curve for the constant-rate birth-death simulations (top left) and increasing deviation from the log-linear curve with higher correlation factor β. Higher standard deviation σ in the speciation rates led to slightly more variation in LTT curves but no large effect.

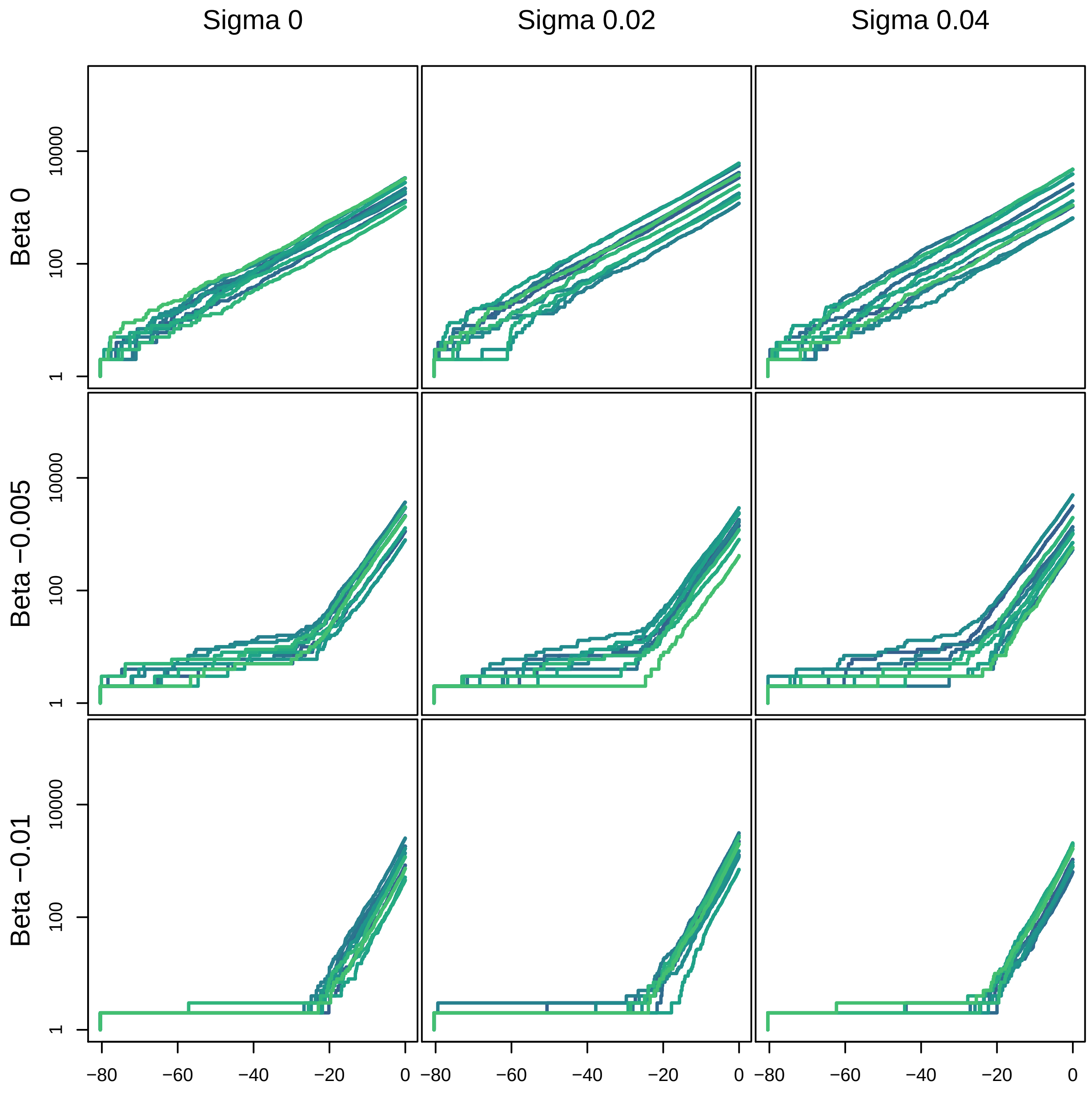

Figure S19: Lineage-through-time (LTT) curves of 10 phylogenies simulated under an environmentally-dependent UC diversification model. We used the simulated diversification rate trajectories shown and described in Figure S17. We nicely observe the log-linear LTT curve for the constant-rate birth-death simulations (top left) and increasing deviation from the log-linear curve with higher correlation factor β. Higher standard deviation σ in the speciation rates led to slightly more variation in LTT curves but no large effect.

S18.3 Estimated correlation factors from the phylogenies simulated under environmentally-dependent diversification rates

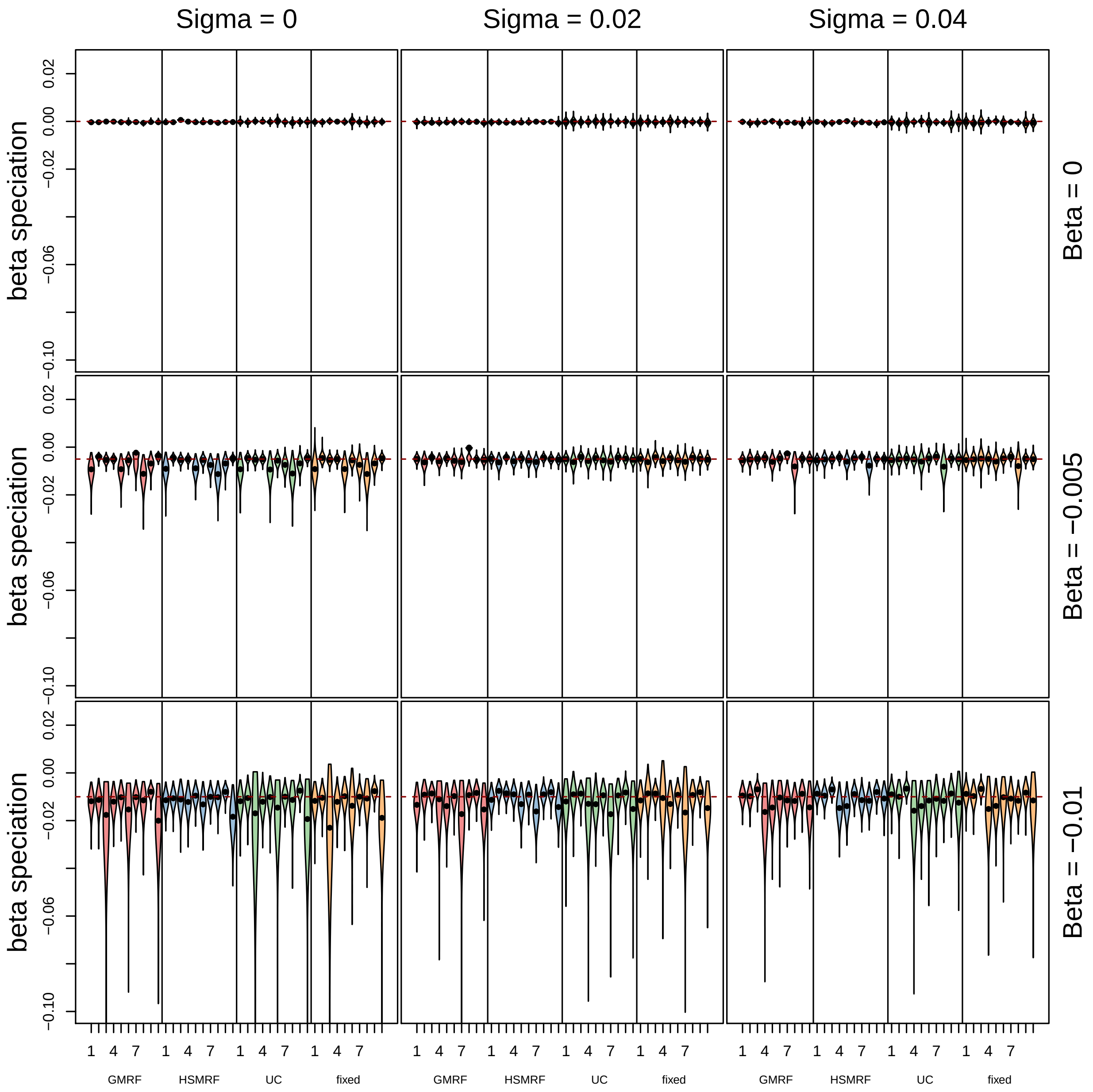

Figure S20: Estimated correlation factors for the simulated phylogenies under the GMRF environmentally-dependent diversification rates model. We estimated correlation factor β using the simulated phylogenies shown and described in Figure S18. We used all four environmentally-dependent diversification rates model for inference, running in total 450 MCMC analyses. Overall, all four models perform well to estimate the environmental correlation. As we noticed in our simulations, the diversification rate variation is primarily driven by the environmental variable and less by additional factors (comparably low σ). In these situations it does not matter much which environmentally-dependent diversification rates model is used. Nevertheless, the HSRMF model always produced the smallest credible intervals and was most precise. This simulation study shows that there is high power to detect environmental correlation to diversification rates (middle and bottom row) while retaining a low false-positive rate (top row). The results are congruent with our inferences using the simulated trees under the UC environmentally-dependent diversification rates model (Figure **??**).

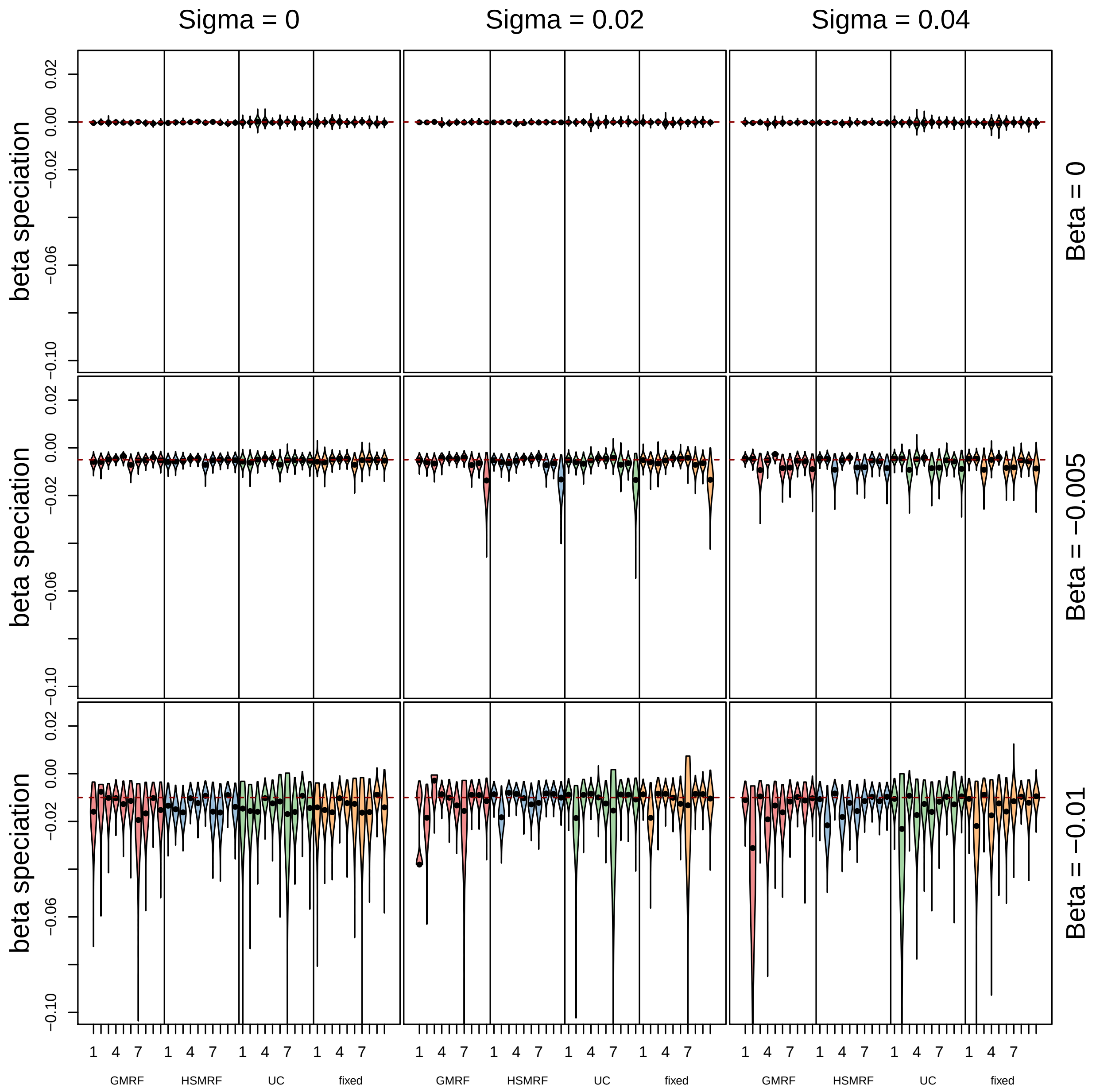

Figure S21: Estimated correlation factors for the simulated phylogenies under the UC environmentally-dependent diversification rates model. We estimated correlation factor β using the simulated phylogenies shown and described in Figure S19. We used all four environmentally-dependent diversification rates model for inference, running in total 450 MCMC analyses. Overall, all four models perform well to estimate the environmental correlation. As we noticed in our simulations, the diversification rate variation is primarily driven by the environmental variable and less by additional factors (comparably low σ). In these situations it does not matter much which environmentally-dependent diversification rates model is used. Nevertheless, the HSRMF model always produced the smallest credible intervals and was most precise. This simulation study shows that there is high power to detect environmental correlation to diversification rates (middle and bottom row) while retaining a low false-positive rate (top row). The results are congruent with our inferences using the simulated trees under the GMRF environmentally-dependent diversification rates model (Figure S21).

S19 Simulation study using empirical taxon sampling

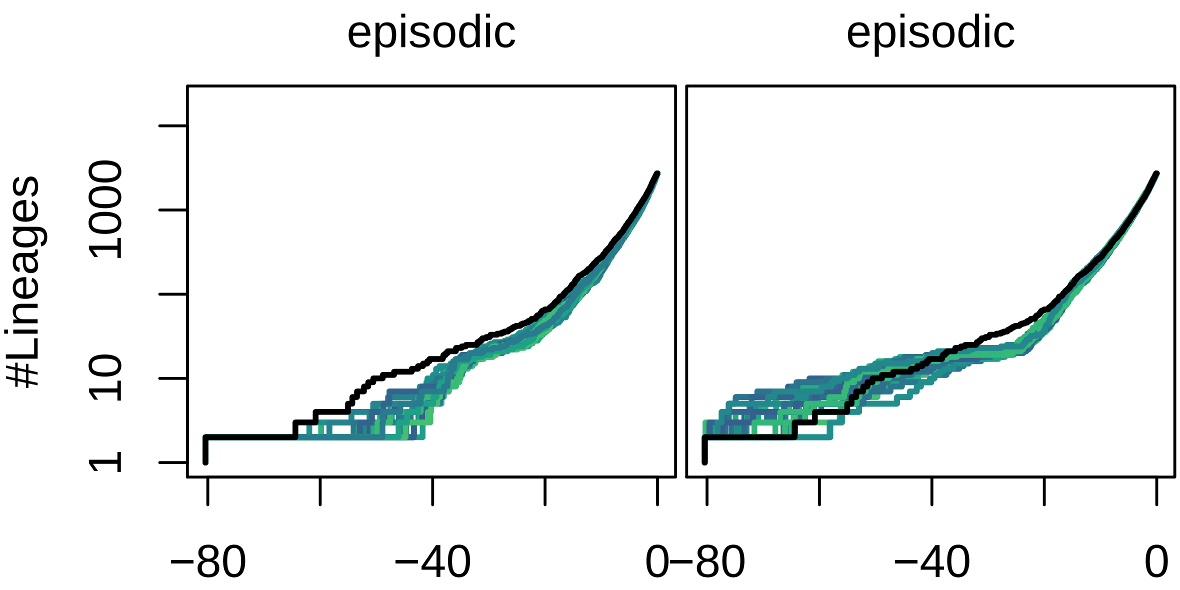

Figure S22: Lineage-through-time curves of simulated phylogenies under empirical sampling. We simulated 100 phylogenies (only first 10 shown here) under a constant-rate birth-death process (false-positive) and an episodic-birth-death process (power) using empirical taxon sampling. These trees were simulated by adding the missing species randomly in the pre-assigned clades of the daisy phylogeny. Then, we simulated divergence times under either the constant or episodic birth-death process. After simulation, the additional taxa were pruned again to mimic empirical sampling. The solid black line shows the empirical LTT curve of the daisy phylogeny.

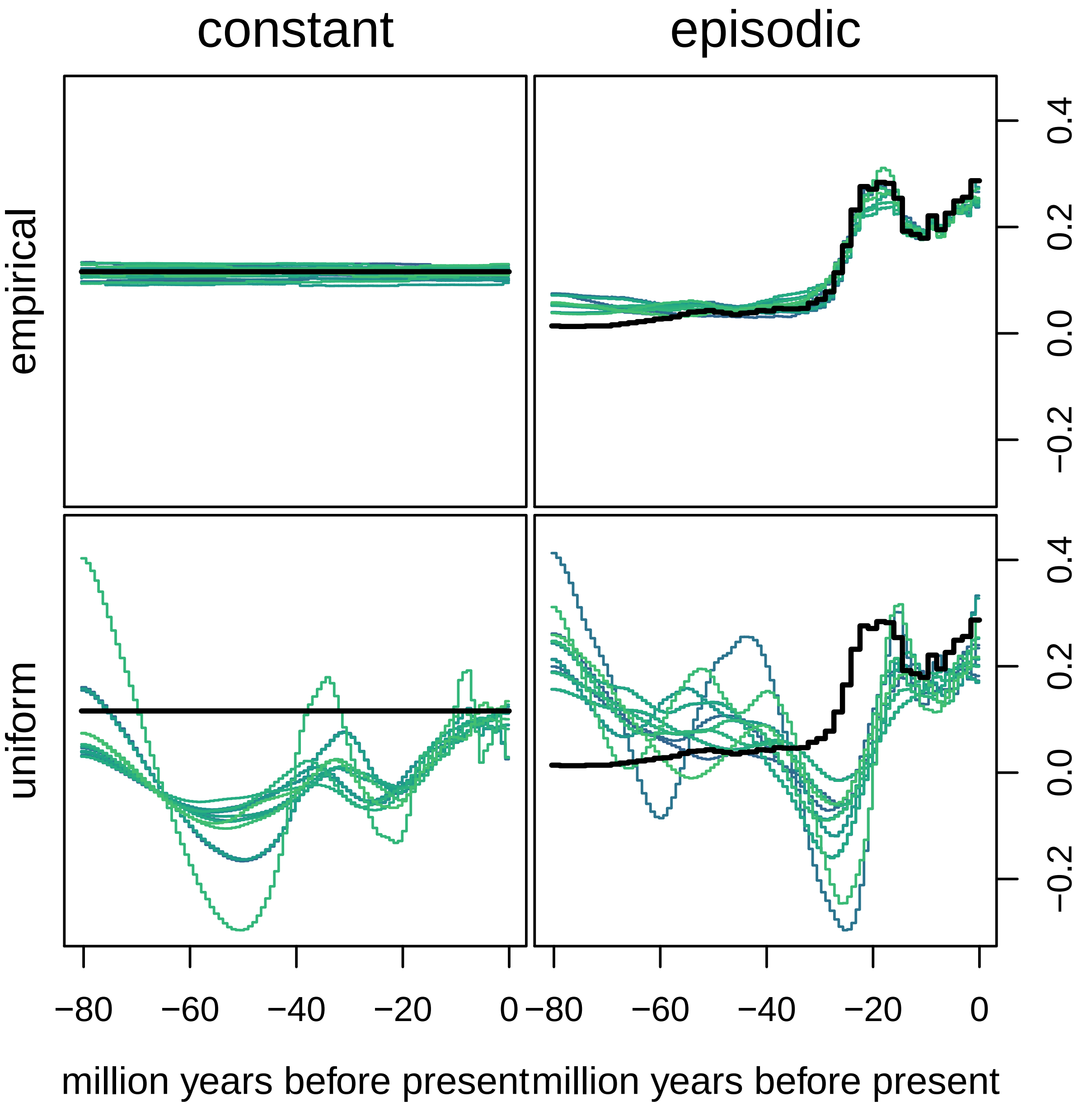

Figure S23: Estimated net-diversification rates using the simulated phylogenies from Figure S22. The solid black line shows our true net-diversification rates. If our assumption of incomplete taxon sampling matches the simulation conditions, then we infer unbiased diversification rates, as previously shown under similar incomplete taxon sampling schemes [11, 10, 18]. Conversely, if the incomplete taxon sampling assumption is violated, e.g., assuming all missing species are *uniformly* distributed within the phylogeny, then we obtain strongly biased diversification rates. We also note that our GMRF model shows a good performance even when the true diversification rates are constant (top left, low false-positive rate). Our good precision in not surprising because the simulated phylogenies had 2723 tips, as our daisy phylogeny.

References

[1] Viviana D Barreda, Eduardo G Ottone, Federico M Dávila, and Ricardo A Astini. Edad y paleoambiente de la formación del buey (mioceno), sierra de famatina, la rioja, argentina: evidencias sedimentológicas y palinológicas. *Ameghiniana*, 43(1):215–226, 2006.

[2] Viviana D. Barreda, Luis Palazzesi, Liliana Katinas, Jorge V. Crisci, María C. Tellería, Kåre Bremer, Mauro G. Passala, Florencia Bechis, and Rodolfo Corsolini. An extinct Eocene taxon of the daisy family (Asteraceae): Evolutionary, ecological and biogeographical implications. *Annals of Botany*, 109(1):127–134, 2012.

[3] Viviana D Barreda, Luis Palazzesi, Maria C Tellería, Eduardo B Olivero, J Ian Raine, and Félix Forest. Early evolution of the angiosperm clade Asteraceae in the Cretaceous of Antarctica. *Proceedings of the National Academy of Sciences*, 112(35):10989–10994, 2015.

[4] S Blackmore, E Van Campo, and PR Crane. Lophate compositae pollen from the miocene and pliocene of the mediterranean region. *Pollen et spores*, 28(3-4):391–402, 1986.

[5] Scott Chamberlain. rgbif: Interface to the Global 'Biodiversity' Information Facility 'API', 2016.

[6] Fabien L. Condamine, Jonathan Rolland, and Hélène Morlon. Macroevolutionary perspectives to environmental change. *Ecology Letters*, 16(SUPPL.1):72–85, 2013.

[7] RA Couper and WF Harris. Pliocene and pleistocene plant microfossils from drillholes near frankton, new zealand. *New Zealand journal of geology and geophysics*, 3(1):15–22, 1960.

[8] ALAN Graham. A contribution to the geologic history of the compositae. In *Compositae: systematics. Proceedings of the international Compositae conference, Kew*, volume 1, pages 123–140. Royal Botanic Gardens Kew, 1994.

[9] Sandy P. Harrison and Colin I. Prentice. Climate and CO2 controls on global vegetation distribution at the last glacial maximum: Analysis based on palaeovegetation data, biome modelling and palaeoclimate simulations. *Global Change Biology*, 9(7):983–1004, jul 2003.

[10] Sebastian Höhna. Likelihood inference of non-constant diversification rates with incomplete taxon sampling. *PLoS ONE*, 9(1):e84184, 2014.

[11] Sebastian Höhna, Tanja Stadler, Fredrik Ronquist, and Tom Britton. Inferring speciation and extinction rates under different sampling schemes. *Molecular Biology and Evolution*, 28(9):2577–2589, 2011.

[12] R.E. Kass and A.E. Raftery. Bayes factors. 90:773–795, 1995.

[13] Brandon Legried and Jonathan Terhorst. A class of identifiable birth-death models. *bioRxiv*, 2021.

[14] EB Leopold. Late cenozoic palynology: in aspects of palynology, rh tschudy and ra scott, 1969.

[15] Andrew F. Magee, Sebastian Höhna, Tetyana I. Vasylyeva, Adam D. Leaché, and Vladimir N. Minin. Locally adaptive Bayesian birth-death model successfully detects slow and rapid rate shifts. *PLoS Computational Biology*, 16(10):e1007999, 2020.

[16] James Ian Raine, DC Mildenhall, and Elizabeth Kennedy. New zealand fossil spores and pollen: an illustrated catalogue, 2011.

[17] Elizabeth L. Spriggs, Pascal-Antoine Christin, and Erika J. Edwards. C4 photosynthesis promoted species diversification during the miocene grassland expansion. *PLoS ONE*, 9(8):e105923, 2014.

[18] Tanja Stadler and Folmer Bokma. Estimating speciation and extinction rates for phylogenies of higher taxa. *Systematic Biology*, 62(2):220–230, 2013.

[19] Wei-Ming Wang. On the origin and development of artemisia (asteraceae) in the geological past. *Botanical Journal of the Linnean Society*, 145(3):331–336, 2004.
