# Supplementary material for: The rise of grasslands is linked to atmospheric CO2 decline in the late Palaeogene
Source: Nat Commun. 2022 Jan 12;13:293. doi: 10.1038/s41467-021-27897-y (PMC8755714; doi:10.1038/s41467-021-27897-y)
Supplement: Supplementary file 3 — Description of Additional Supplementary Files [file 41467_2021_27897_MOESM3_ESM.docx]

Description of Additional Supplementary Files

Title: Supplementary Data 1

Description: Time-calibrated phylogeny of Asteraceae
