# Supplementary material for: The rise of grasslands is linked to atmospheric CO2 decline in the late Palaeogene
Source: Nat Commun. 2022 Jan 12;13:293. doi: 10.1038/s41467-021-27897-y (PMC8755714; doi:10.1038/s41467-021-27897-y)
Supplement: Supplementary file 5 — Reporting Summary [file 41467_2021_27897_MOESM5_ESM.pdf]

nature portfolio

Corresponding author(s): Luis Palazzesi

Last updated by author(s): Nov 28th 2021

Reporting Summary

Nature Portfolio wishes to improve the reproducibility of the work that we publish. This form provides structure for consistency and transparency in reporting. For further information on Nature Portfolio policies, see our Editorial Policies and the Editorial Policy Checklist.

Statistics

For all statistical analyses, confirm that the following items are present in the figure legend, table legend, main text, or Methods section.

n/a	Confirmed	
☐	☒	The exact sample size (*n*) for each experimental group/condition, given as a discrete number and unit of measurement
☒	☐	A statement on whether measurements were taken from distinct samples or whether the same sample was measured repeatedly
☒	☐	The statistical test(s) used AND whether they are one- or two-sided<br>*Only common tests should be described solely by name; describe more complex techniques in the Methods section.*
☒	☐	A description of all covariates tested
☒	☐	A description of any assumptions or corrections, such as tests of normality and adjustment for multiple comparisons
☐	☒	A full description of the statistical parameters including central tendency (e.g. means) or other basic estimates (e.g. regression coefficient) AND variation (e.g. standard deviation) or associated estimates of uncertainty (e.g. confidence intervals)
☒	☐	For null hypothesis testing, the test statistic (e.g. *F*, *t*, *r*) with confidence intervals, effect sizes, degrees of freedom and *P* value noted<br>*Give P values as exact values whenever suitable.*
☐	☒	For Bayesian analysis, information on the choice of priors and Markov chain Monte Carlo settings
☐	☒	For hierarchical and complex designs, identification of the appropriate level for tests and full reporting of outcomes
☒	☐	Estimates of effect sizes (e.g. Cohen's *d*, Pearson's *r*), indicating how they were calculated

Our web collection on statistics for biologists contains articles on many of the points above.

Software and code

Policy information about availability of computer code

Data collection	We used NCBIminer tool v2.0 and rgbif v3.0 for data collection.
Data analysis	We used TESS v2.1.0; RevBayes v1.1.1; R v4.0.0.; Phytools v0.7; ggtree v1.14 for simulations, diversification analyses, and plots.<br>All tutorials to conduct the analysis are deposited in https://revbayes.github.io/tutorials/

For manuscripts utilizing custom algorithms or software that are central to the research but not yet described in published literature, software must be made available to editors and reviewers. We strongly encourage code deposition in a community repository (e.g. GitHub). See the Nature Portfolio guidelines for submitting code & software for further information.

Data

Policy information about availability of data

All manuscripts must include a data availability statement. This statement should provide the following information, where applicable:

- Accession codes, unique identifiers, or web links for publicly available datasets
- A description of any restrictions on data availability
- For clinical datasets or third party data, please ensure that the statement adheres to our policy

The authors declare that all data supporting the findings of this study are available within the article and its supplementary information files or can be requested to authors. For the new models and analyses in this paper, we have created specific tutorials which we host and maintain on our RevBayes website: 1) Estimating time varying diversification rates using the GMRF and HSMRF models: https://revbayes.github.io/tutorials/divrate/ebd.html 1) Estimating environmentally-dependent diversification rates: https://revbayes.github.io/tutorials/divrate/env.htm1 3) Incorporating incomplete taxon sampling using either uniform or empirical taxon sampling: https://revbayes.github.io/tutorials/divrate/sampling.html

Field-specific reporting

Please select the one below that is the best fit for your research. If you are not sure, read the appropriate sections before making your selection.

☐ Life sciences ☐ Behavioural & social sciences ☒ Ecological, evolutionary & environmental sciences

For a reference copy of the document with all sections, see nature.com/documents/nr-reporting-summary-flat.pdf

Ecological, evolutionary & environmental sciences study design

All studies must disclose on these points even when the disclosure is negative.

Study description	We described new computational model to test diversifications rates through time using sampled phylogenies.
Research sample	We selected 2,723 species of Asteraceae to build a phylogenetic tree, including about 10% of the total diversity of the family. The source come from molecular datasets retrieved using NBCIminer.
Sampling strategy	Sample size was not determined statistically. There was no sampling strategy, although we included several species for each subfamily so every major taxonomic group is represented.
Data collection	We collected data using NCBIminer to recover molecular data from GenBank. No DNA sequences were generated in this study.
Timing and spatial scale	The timing and spatial scale was not relevant to our study as it does not include any field work or biological experiment.
Data exclusions	No data were excluded from the Analysis.
Reproducibility	Any experiment can be fully reproduced using our tutorials.
Randomization	Randomization was not relevant to our study because our research does not include any biological experiment.
Blinding	Blinding was nor relevant to our study because our study does not include any clinical research study.

Did the study involve field work? ☐ Yes ☒ No

Reporting for specific materials, systems and methods

We require information from authors about some types of materials, experimental systems and methods used in many studies. Here, indicate whether each material, system or method listed is relevant to your study. If you are not sure if a list item applies to your research, read the appropriate section before selecting a response.

Materials & experimental systems

n/a	Involved in the study
☒	☐ Antibodies
☒	☐ Eukaryotic cell lines
☒	☐ Palaeontology and archaeology
☒	☐ Animals and other organisms
☒	☐ Human research participants
☒	☐ Clinical data
☒	☐ Dual use research of concern

Methods

n/a	Involved in the study
☒	☐ ChIP-seq
☒	☐ Flow cytometry
☒	☐ MRI-based neuroimaging
